# Natural Variation in Ovule Morphology Is Influenced by Multiple Tissues and Impacts Downstream Grain Development in Barley (*Hordeum vulgare* L.)

**DOI:** 10.3389/fpls.2019.01374

**Published:** 2019-10-31

**Authors:** Laura G. Wilkinson, Xiujuan Yang, Rachel A. Burton, Tobias Würschum, Matthew R. Tucker

**Affiliations:** ^1^School of Agriculture, Food and Wine, University of Adelaide, Urrbrae, SA, Australia; ^2^State Plant Breeding Institute, University of Hohenheim, Stuttgart, Germany

**Keywords:** barley, ovule, nucellus, grain, pistil, yield

## Abstract

The ovule plays a critical role in cereal yield as it is the site of fertilization and the progenitor of the grain. The ovule primordium is generally comprised of three domains, the funiculus, chalaza, and nucellus, which give rise to distinct tissues including the integuments, nucellar projection, and embryo sac. The size and arrangement of these domains varies significantly between model eudicots, such as *Arabidopsis thaliana*, and agriculturally important monocotyledonous cereal species, such as *Hordeum vulgare* (barley). However, the amount of variation in ovule development among genotypes of a single species, and its functional significance, remains unclear. To address this, wholemount clearing was used to examine the details of ovule development in barley. Nine sporophytic and gametophytic features were examined at ovule maturity in a panel of 150 European two-row spring barley genotypes, and compared with grain traits from the preceding and same generation. Correlations were identified between ovule traits and features of grain they produced, which in general highlighted a negative correlation between nucellus area, ovule area, and grain weight. We speculate that the amount of ovule tissue, particularly the size of the nucellus, may affect the timing of maternal resource allocation to the fertilized embryo sac, thereby influencing subsequent grain development.

## Introduction

Barley is a cereal that has sustained humans for thousands of years and remains a crop of key agricultural and economic importance (Samuel 1996; Eitam et al., 2015). A large portion of the economic value of barley comes from the endosperm of the grain, which is a key source of calories for direct consumption by livestock and humans (Sands et al., 2009), as well as a source of protein and fermentable sugars for the malting and brewing industries (Gupta et al., 2010). The effects of climate change are predicted to negatively impact global barley yield over the next 50 years, and as such, efforts have been directed towards breeding elite barley genotypes with higher yield and robust tolerance to environmental stress (Tester and Langridge 2010).

Before grain can be produced, barley plants must generate floral heads, also referred to as inflorescences or spikes. In two-rowed barley, the central rachis of each spike is flanked by individual spikelets (flowers), which contain multiple florets of which only the central floret is fertile. All of the organs and tissues required for self-fertilization and seed production are located within this floret. This includes a single ovule within a single ovary (pistil), which together comprise the female reproductive organs that give rise to and protect the bulk of the tissues within the grain. The number of mature florets has been linked to barley yield (Alqudah and Schnurbusch, 2014), while the size and number of cells within the pistil has been linked to spike dry weight in wheat (Guo et al., 2015; Guo et al., 2016). Heat and drought stress have been shown to compromise aspects of pistil and ovule maturation, leading to defects in fertilization and grain development in wheat (*Triticum aestivum*) and maize (*Zea mays*; (Saini et al., 1983;Jäger et al., 2008; Oury et al., 2016; Onyemaobi et al., 2018). Thus, correct development of the female reproductive organs is an important determinant of floral fertility and yield, especially under conditions of environmental stress. A greater understanding of ovule and pistil development in cereal crops may provide breeding targets for improved yield and yield stability.

The ovule establishes the basic framework for seed production and its development has been heavily studied in a range of species including *Arabidopsis* (Schneitz et al., 1995; Pinto et al., 2019) and rice (Itoh et al., 2005; Colombo et al., 2008), highlighting similarities in tissue development and function. In general, ovule primordia consist of three domains including a proximal connective tissue (the funiculus), central chalaza, and distal nucellus (Schneitz et al., 1995). The funiculus acts as a stalk to connect the ovule to the maternal plant in *Arabidopsis*, but is absent in cereal species (Engell 1994; Maheswari 1950). Instead, this role is fulfilled by the chalaza, which connects the ovule to the ovary and also gives rise to the integuments that surround the nucellus. The nucellus is located at the distal tip of the ovule and gives rise to the megasporocyte and embryo sac (germline; Shirley et al., 2018; Pinto et al., 2019). Similar to *Arabidopsis*, embryo sac development in monocotyledonous cereals such as barley follows the *Polygonum*-type (Willemse and van Went, 1984; Schneitz et al., 1995). However, barley ovules are much larger than those of *Arabidopsis* due to a multilayered nucellus that contributes up to 65% of the ovule area at maturity (Wilkinson and Tucker 2017). In general, cereal ovules are described as crassinucellar, meaning the megasporocyte is separated from the ovule epidermis by at least two cell layers (Wilkinson et al., 2018). This differs from *Arabidopsis* that produces a small tenuinucellate nucellus dominated by a megasporocyte that directly adjoins the nucellar epidermis. In barley, the nucellus appears intermediate between the two forms in that it is large and multilayered but tenuinucellate during early development (Bennett et al., 1973; Engell, 1989). Despite these differences, in both *Arabidopsis* and barley the nucellus gives rise to a single megasporocyte (megaspore mother cell) that undergoes meiosis to produce a tetrad of four reduced megaspores (megasporogenesis), one of which is selected to become the functional megaspore and initiate embryo sac development (megagametogenesis). In both barley and *Arabidopsis* the embryo sac consists of seven different cell types with discrete functions. The position of the egg apparatus, central cell, and antipodal cells are conserved, but the number of antipodal cells varies considerably from three in *Arabidopsis* to at least 30 in barley and other cereals (Brink and Cooper 1944; Diboll 1968;Chaban et al., 2011).

Different ovule tissues play distinct roles in downstream seed development. For example, the integuments differentiate into a seed coat that provides physical protection for the seed (Debeaujon et al., 2000), while the embryo sac gives rise to the endosperm and embryo after fertilization of the central cell and egg cell respectively. Genes that influence integument growth or endosperm divisions in *Arabidopsis* have been implicated in the control of seed size (Garcia et al., 2005; Ingouff et al., 2006; Adamski et al., 2009; Batista et al, 2019), suggesting a role for both maternal and filial factors in the control of seed development. The role of the nucellus in downstream seed development is less clear, but is likely to fulfill distinct functions depending on the species. In *Arabidopsis*, the nucellus gives rise to the female germline before diminishing during subsequent pre-fertilization stages (Xu et al., 2016). In barley and wheat, similar to *Arabidopsis*, the nucellus gives rise to the germline. However, it subsequently increases in size and only diminishes after fertilization, when constituent cells undergo programmed cell death (PCD) and differentiate to form the nucellar projection (Dominguez et al., 2001; Thiel et al., 2008). This tissue fulfills a key role in funneling maternal nutrients into the endosperm through the endosperm transfer cells. Defects in differentiation of the nucellar projection, through down-regulation of the *Jekyll* gene for example, result in severe defects in grain fill (Radchuk et al., 2006). Previous studies in a small panel of barley genotypes suggested that nucellus size varies at ovule maturity, although the effect on grain development was not determined (Wilkinson and Tucker 2017). One possibility is that remobilization of reserves *via* nucellar PCD provides a local nutrient source for early endosperm development, and hence variation in nucellus size might impact key features of seed size and morphology.

This study aimed to define the bounds of normal ovule morphology in a population of 150 two-row spring barley genotypes, and investigate possible correlations between ovule morphology and mature grain traits. Correlations were identified between different ovule tissues, revealing that the nucellus and embryo sac both contribute to overall ovule size but in a genotype-dependent manner. Small but significant correlations were identified between mature ovule features and the grain they produced, and suggest that increased ovule and nucellus size may have a negative impact on grain size and weight.

## Methods and Materials

### Plant Growth

A panel of 150 European two-row spring barley genotypes, representing a sub-panel of the genotypes described by Comadran et al. (2012) that show limited population structure and similar flowering time, were sourced from the James Hutton Institute, Scotland. In 2014, plants were grown in one large glasshouse at The Plant Accelerator, Adelaide, Australia, in a 50:50 cocopeat:clay-loam soil mixture (v/v), under a 22/15°C day/night temperature regime with natural light conditions. Seeds were sown in May and harvested in September, at a density of one plant per pot. Harvested grain were hand-threshed, analyzed, and re-sown in 2015. The resulting plants were grown in two small glasshouses, in the same soil mixture, and under the same temperature regime, but with different day length due to later sowing (July). Plants were grown in triplicate with a randomized pot sequence. Pistils were harvested from all plants as described below and phenotypes were compared between the replicate samples in the two glasshouses (GH14 and GH17). Statistical analysis suggested there was a small but significant difference in mature ovule measurements; for example, the average ovule area in GH14 was 170,323.42 µm^2^ (n=573) compared to 176,697.89 µm^2^ (n = 457) in GH17 (*t*-test, p = 0.001; **Figure S1A**). Ratios of the GH14 to GH17 measurements varied from 0.96 to 1.0 and suggested that most features were slightly smaller in GH14 (**Figure S1B**). Because ovules from all genotypes were sampled from both glasshouses, average values were used for all subsequent analysis. In September 2016, a selection of 10 genotypes (Cecilia, Forum, Gant, Akita, Optic, Host, Foxtrot, Wren, Salka, and Lina) were re-grown to assess whether differences in ovule measurements were reproducible.

### Sample Collection and Microscopy

Pistils were dissected from anthesis stage florets based on physical appearance similar to stage 9.5 of the Waddington Scale (Waddington et al., 1983), and by the presence of bright yellow anthers that only released pollen when gently crushed. At least three pistils (five at most) were hand dissected from the middle of one inflorescence of all three replicates of each genotype, where possible. After clearing (see below), damaged or insufficiently cleared samples were discarded, eventually leaving between 4 and 15 mature ovules (stage Ov10; see **Figure S2**) for analysis per genotype.

**Figure 1:**
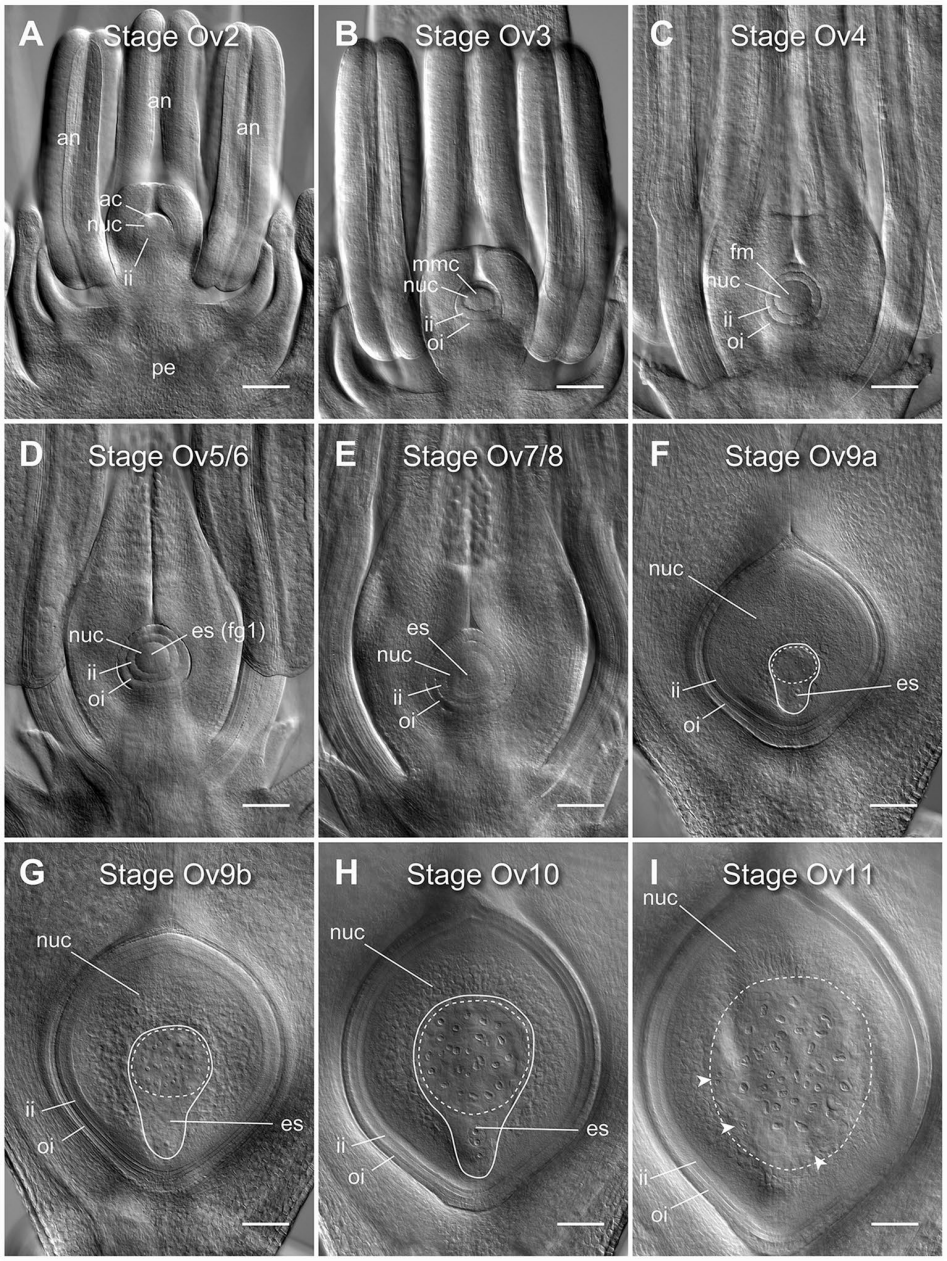
Clearing of whole barley florets and pistils reveals different stages of ovule development. **(A)** At stage Ov2 the ovule has initiated in the center of the carpel and is characterized by the selection of an archesporial cell and initiation of the inner integument. **(B)** At stage Ov3 the megaspore mother cell (MMC) has differentiated from the archesporial cell and both integuments are observed. **(C)** At stage Ov4 the MMC has undergone meiosis, giving rise to four haploid daughter cells. The nucellar dome is obvious between the two growing integuments. **(D)** At stage Ov5/6 the functional megaspore has been selected and possibly initiated mitosis to form the embryo sac (ES), while the nucellar dome is not fully enclosed. **(E)** At stage Ov7/8 the integument has closed over the nucellus thus forming the micropyle. During this stage, the embryo sac will complete mitotic divisions and cellularize, producing two synergid cells, an egg cell, a central cell with two polar nuclei, and at least three antipodal cells. **(F)** At stage Ov9a growth of the ovule has increased rapidly, and the antipodal cells proliferate to become a group of 15 to 45 small and tightly clustered cells. **(G)** At stage Ov9b growth of the ovule has begun to slow, and the antipodal cells are distinctly less tightly clustered. **(H)** At stage Ov10 the ovule reaches anthesis, or reproductive maturity, discernible from stage 9b by slightly larger nucellus and embryo sac areas, and greater spacing of the antipodal cell nuclei. **(I)** At stage Ov11 the ovule is fertilized, as determined by a combination of a large increase in ovule size, visibility of sperm nuclei, lack of visibility of the polar nuclei, irregular shapes of antipodal cells, and clusters of small nuclei at the periphery of the embryo sac. ac, archesporial cell; es, embryo sac; fg1, one-cell female gametophyte; fm, functional megaspore; ii, inner integument; mmc, megaspore mother cell; oi, outer integument; nuc, nucellus. A solid line indicates the bounds of the embryo sac, a dashed line indicates the bounds of the antipodal cell cluster, arrowheads indicate additional clusters of nuclei after fertilization. Images from the genotype Salka. Scale bars = 100 µm.

In order to assess earlier stages of ovule development, the Waddington Scale (Waddington et al., 1983) was again used to stage florets during spike development. Five florets were collected from the middle of at least three inflorescences from Waddington stage 6 until fertilization. At stages prior to Waddington stage 8.5 whole florets were collected; for samples after this stage only the pistil was collected. Pre-anthesis samples were analyzed in 10 genotypes showing diverse morphology at anthesis, including Cecilia, Forum, Gant, Akita, Optic, Host, Foxtrot, Wren, Salka, and Lina. These stages are represented in **Figure 1** and **Figure S2** by the Salka genotype.

### Clearing and Microscopy

Pistils were fixed in FAA (10% formalin, 5% glacial acetic acid, 50% ethanol, 35% millipore H_2_O, plus a drop of Triton X100) overnight, then dehydrated through an ethanol series (3 × 30 min at each step of 70, 80, 90, 95, 100%) and placed into Hoyer’s solution as described in Wilkinson and Tucker (2017). Ovules within the cleared pistil tissue were observed using differential interference contrast (DIC) microscopy and Nomarski prisms on a Zeiss Axio Imager M2, and captured as z-stack images encompassing the entire ovule from dorsal to ventral aspect in 40 optical sections. Composite images for figures were assembled in Adobe Photoshop and Illustrator (both version CC 2018; Adobe Inc., USA).

### Quantitative Analysis of Mature Ovule Morphology

Nine morphological traits were measured from the z-stack images, using Zeiss Zen Blue (2012) software as described in Wilkinson and Tucker (2017). Each trait represents a one- or two-dimensional measurement, and data reflects the widest point of the region of interest visible within the z-stack. The nine measurements collected were: ovule area (OV_A), ovule transverse width (OV_T), ovule longitudinal height (OV_L), embryo sac area (ES_A), embryo sac transverse width (ES_T), embryo sac longitudinal height (ES_L), nucellus area (NUC_A), nucellus proportion (NUC_P), and integument width (INT_W). Measurements were averaged from between 4 and 15 anthesis stage ovules (stage Ov10), representing at least two of the three replicate plants from each genotype. Low sample numbers (< 4 ovules) due to poor plant health, insufficient clearing, tissue damage, or staging errors resulted in elimination of 23 genotypes from the analysis, reducing the initial population of 150 genotypes to a functional population of 127. Integument “area” was not measured due to difficulties in accurately scoring the boundaries. Thus, what is presented as ovule area is essentially nucellus plus embryo sac area.

### Data Analysis

Genotypic variance components were obtained from linear mixed models with a random genotypic effect and their significance was tested by model comparison with likelihood ratio tests where the halved P values were used as an approximation (Stram and Lee 1994). Repeatability (R) was estimated from these models following the approach suggested by Piepho and Möhring (2007). Trait correlations, dendrograms, and principal component analyses were performed using default parameters in the “corrplot” package (https://cran.r-project.org/web/packages/corrplot/corrplot.pdf) in R with RStudio (R version 3.5.0; RStudio^®^, USA). Unless indicated otherwise, Pearson’s correlation coefficients and associated significance values are shown. Figures were assembled in Adobe Illustrator CC 2018 (version 22.0.0, Adobe Inc., USA).

### Grain Trait Measurements

Grain traits were analyzed using a SeedCount^™^ SC4 (Seed Count Australasia, Condell Park, Australia) at the University of Adelaide, following manufacturer’s instructions. Data were obtained from two generations of the same genotypes i.e. grain that was sown and collected in 2014 (“2014” grain), and grain that sown and collected in 2015 (“2015” grain). Ovule phenotypes were collected from plants that gave rise to the 2015 grain. In total, sufficient numbers and replicates of grain and ovule data were obtained for 73 genotypes.

## Results

### Progression of Ovule Development in Barley Can Be Tracked by Whole Pistil Clearing

Nine distinct stages of barley ovule development were discernible in cleared floral tissue using DIC microscopy (**Figure 1**). These stages were aligned with ovule staging systems previously reported for rice to aid analysis and cross-species comparisons, and are referred to as stages Ov2 to Ov11 (Lopez-Dee et al., 1999; Itoh et al., 2005). In barley, stages Ov2 to Ov4 encapsulate outgrowth of the initial ovule primordium which includes: integument initiation and archesporial cell differentiation (Ov2), integument outgrowth and megaspore mother cell differentiation (Ov3) and integument “over-growth” and meiosis/megaspore selection (Ov 4) (**Figures 1A–C**; **Figure S2**). Stages Ov5 to Ov8 incorporate the events of embryo sac mitosis. These were difficult to precisely phenotype based only on embryo sac features (**Figures 1D, E**), but other morphological differences were evident. At stage Ov5/6, the nucellar dome was still obvious and ovules showed evidence of functional megaspore expansion (**Figure 1D**), while at stage Ov7/8, the integuments had fully encapsulated the nucellus and the embryo sac contained two to four free nuclei (**Figure 1E**). At stage Ov9a, a fully cellularized embryo sac was present in ovules and antipodal cells had started proliferating, concurrent with massive proliferation/expansion of the nucellus and embryo sac (**Figure 1F**). Antipodal divisions appeared to be complete at stage Ov9b, and the antipodals themselves were less tightly-clustered compared to Ov9a (**Figure 1G**). Ovules at stage Ov10 were fully mature and discernible from Ov9a/b by clear definition of individual antipodal cells that contained enlarged nuclei, a central cell containing two unfused polar nuclei and an egg cell with a prominent nucleus (**Figure 1H**). Stage Ov11 was used to broadly group ovules that had recently been fertilized (**Figure 1I**). This series of ovule stages from Ov2 to Ov10 is equivalent to the phase of barley pistil development represented by stages W6 to W10 on the Waddington scale (**Figure S2**). Ovule maturity (Ov10) appeared to have been reached by W9.5 to W10, just prior to anthesis and “blooming” as described by Brenchley (1920). In the absence of fertilization, only minor changes in ovule morphology could be observed in late stage pistils despite some changes in anther growth and stigma opening, while lodicules were not examined.

### Mature Ovule Morphology Varies Among Two-Row Spring Barley Genotypes

Mature ovule morphology (Ov10) was measured in all 150 genotypes in terms of 2-dimensional area and 1-dimensional distances (Wilkinson and Tucker 2017). Regional areas were measured by following the widest boundary of the tissue of interest at any point within the z-stack. In the majority of genotypes, ovules at maturity exhibited an overall similar appearance including a prominent embryo sac, large antipodal nuclei, and an enlarged central vacuole (**Figures 1H**, **S3B**). Immature ovules were occasionally identified that showed an unusually small antipodal cluster, central cell, and short distance between the micropyle and top of the embryo sac (**Figure S3A**). At the other extreme, fertilized ovules could easily be distinguished by the presence of irregularly shaped antipodal cell nuclei, clusters of small nuclei at the periphery of the embryo sac and a much larger ovule area (**Figures 1I**, **S3C**). The incidence of these “extremes” that were immature or fertilized may reflect sampling error, natural mutants, and/or indicate that reproductive maturity is not perfectly synchronous between the anther and ovule in all barley genotypes. For the purposes of this study, the most dramatic extremes were considered to be incorrectly staged ovules and were not examined further, leaving sufficient data for analysis and comparison of 127 genotypes (**Table S1**).

**Figure 2:**
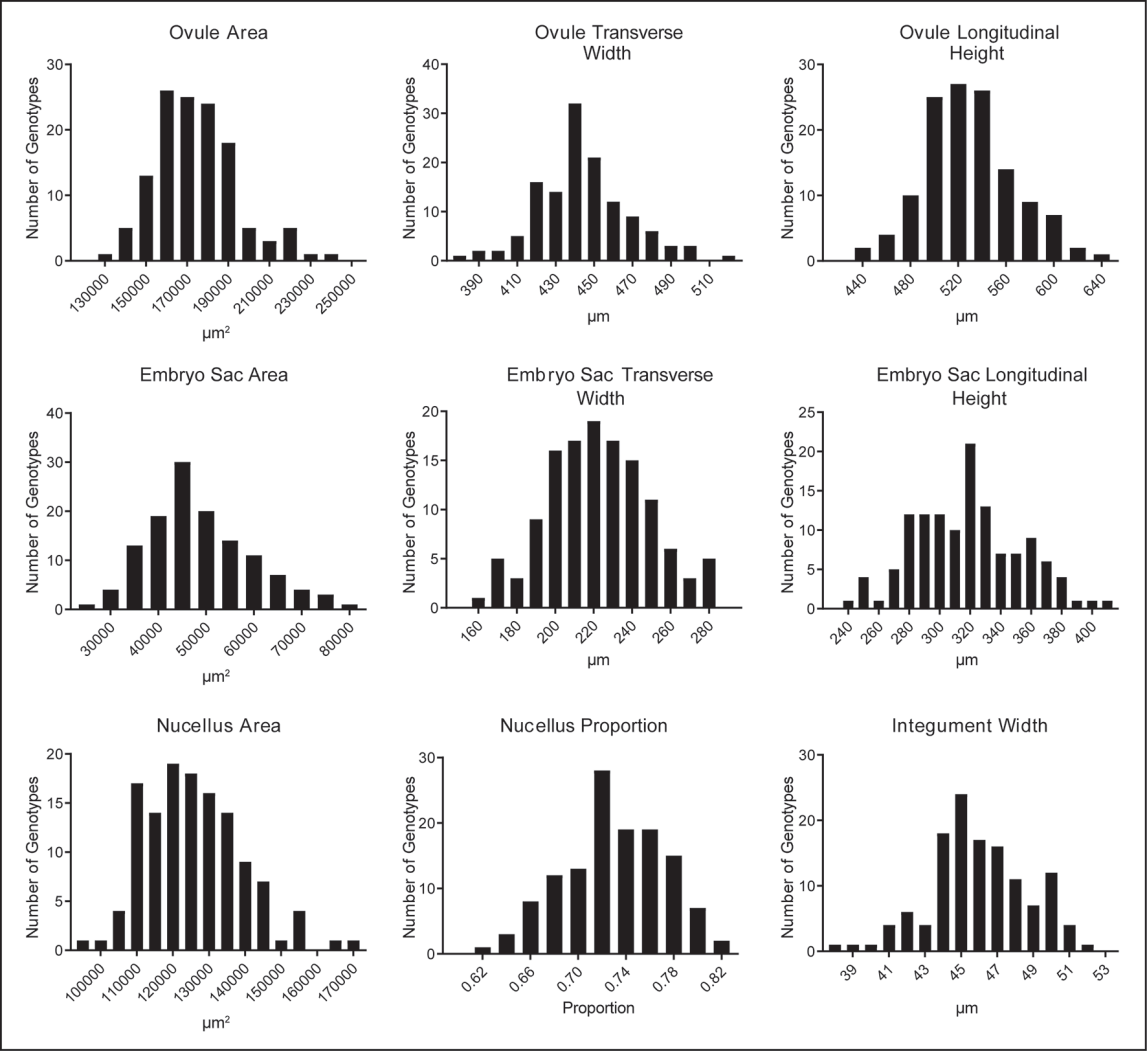
Natural variation in nine mature ovule traits observed in 127 genotypes of European two-row spring barley, represented as frequency distribution plots.

Quantification of ovule morphology revealed natural variation in all traits (**Figure 2**, **Table 1** and **S1**). With the possible exception of integument width, most traits followed a normal distribution. The most variable trait was embryo sac area, with an average size of 48,876.2 ± 10,844.2 µm^2^ and a standard deviation (SD) equating to approximately 22% variation in size. Ovule area and nucellus area were comparatively less variable, observed to be 174,421.2 ± 19,857.8 µm^2^ (11.4% SD) and 125,560.8 ± 13,408.7 µm^2^ (10.7% SD), respectively. Consistent with this, the transverse and longitudinal measurements of the embryo sac varied more than the transverse and longitudinal measurements of the ovule (**Table 1**). Of all traits measured, ovule transverse width was the least variable, followed by integument width and the proportion of nucellus within the ovule (calculated as nucellus area/ovule area). Statistical analysis suggested that the difference between genotypes (i.e. the genotypic variance) was significant for all traits (**Table 1**). Moreover, repeatability (R) estimates were moderate to moderately high, ranging from 0.27 for ovule area to 0.59 for integument width, and sit in the range expected for complex traits based on a single location trial.

**Table 1:** Summary of natural variation, repeatability, and genotypic variance in nine mature ovule traits in 127 genotypes of two-row spring barley. OV_A, ovule area (µm^2^); OV_T, ovule transverse width (µm); OV_L, ovule longitudinal height (µm); ES_A, embryo sac area (µm^2^); ES_T, embryo sac transverse width (µm); ES_L, embryo sac longitudinal height (µm); INT_W, integument width (µm); NUC_A, nucellus area (µm^2^); NUC_P, nucellus proportion (%).

	OV_A	OV_T	OV_L	ES_A	ES_T	ES_L	INT_W	NUC_A	NUC_P
**Average**	174421.2	444.8	529.1	48876.1	222.4	318.0	45.9	125560.8	0.724
**St. Dev.**	19857.8	23.2	38.6	10844.2	26.3	34.2	2.7	13408.7	0.042
**St. Dev. as % of Avg**	11.4	5.2	7.3	22.2	11.8	10.8	5.8	10.7	5.8
**Maximum**	241310.7	519.2	639.8	77888.1	282.0	406.6	52.2	169151.8	82.1
**Minimum**	128009.3	384.0	440.0	25194.4	158.1	238.8	38.1	95875.3	61.1
**Maximum as % of Avg**	138.3	116.7	120.9	159.4	126.8	127.9	113.6	134.7	113.5
**Minimum as % of Avg**	73.4	86.3	83.2	51.5	71.1	75.1	83.0	76.4	84.1
**Repeatability (R)**	0.27	0.27	0.34	0.48	0.46	0.34	0.59	0.31	0.56
**P-value**	0.026	0.019	0.006	<0.001	0.004	0.009	<0.001	0.009	<0.001

**Figure 3:**
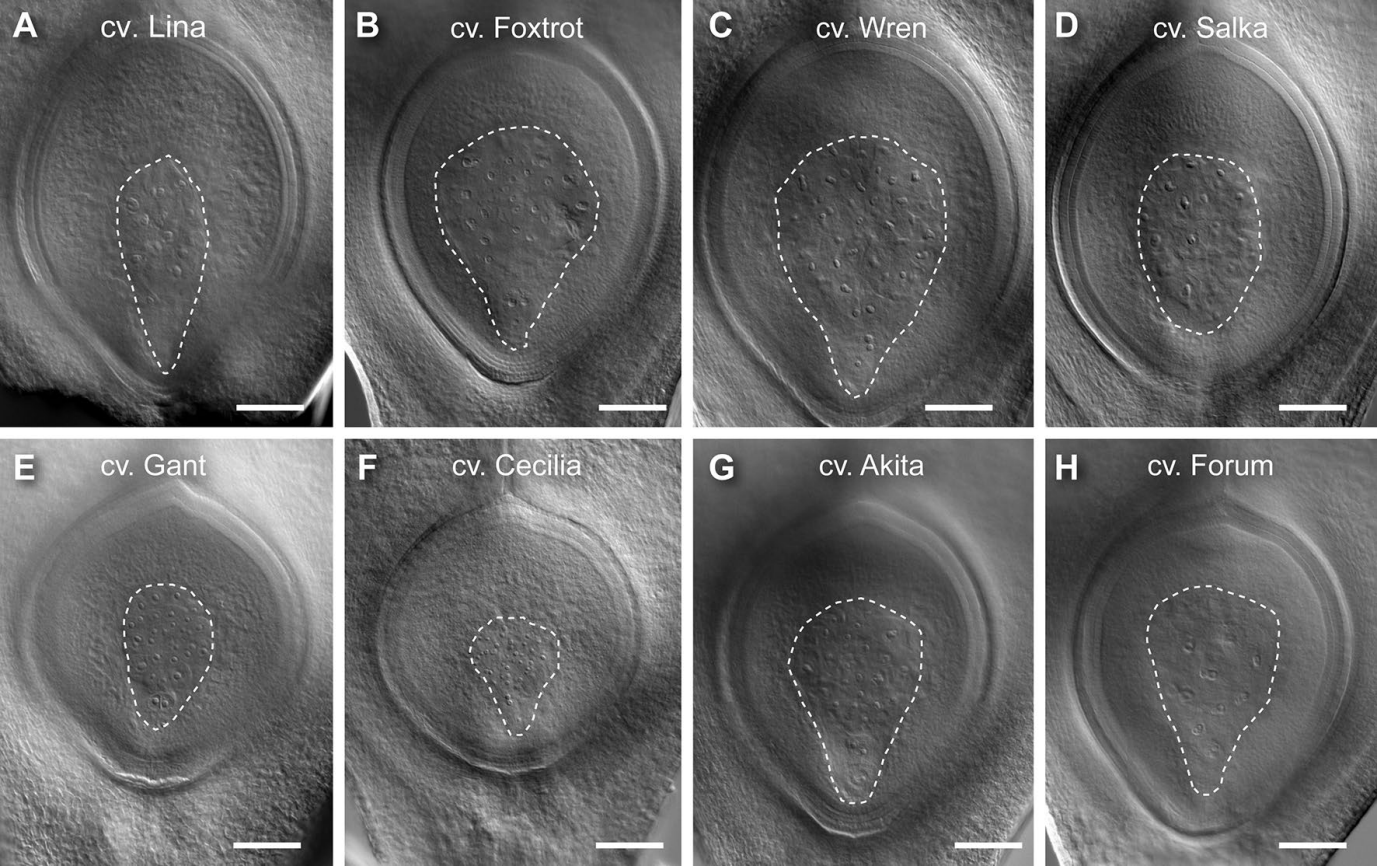
Examples of barley genotypes showing differences in ovule morphology at maturity. **(A)** A representative large ovule in Lina. **(B)** A representative large ovule in Foxtrot. **(C)** A representative large ovule in Wren. **(D)** A representative large ovule in Salka. **(E)** A representative small ovule in Gant. **(F)** A representative small ovule in Cecilia. **(G)** A representative small ovule in Akita. **(H)** A representative small ovule in Forum. Composite images were created by overlaying sequential optical sections from the z-stack. Scale bar = 100 µm.

For all traits, at least 30 genotypes were found to have phenotypic variation that fell outside one SD (**Table 1**, **S2**). Examples of genotypes showing distinct differences are shown in **Figure 3**. For example, Lina, Foxtrot, Wren, and Salka all produced large ovules (**Figures 3A–D**, respectively), with a particularly large nucellus in Lina and Salka. In contrast, Gant, Cecilia, Akita, and Forum produced relatively small ovules (**Figures 3E–H**, respectively), with Akita and Forum producing a relatively small nucellus. These data indicate that the ovule traits under examination in this panel show significant variation between genotypes, and might therefore be used to examine their relationship to each other and the downstream events of seed development.

### Ovule Component Tissues Show Similar Relationships Despite Genotype-Specific Differences

**Figure 4:**
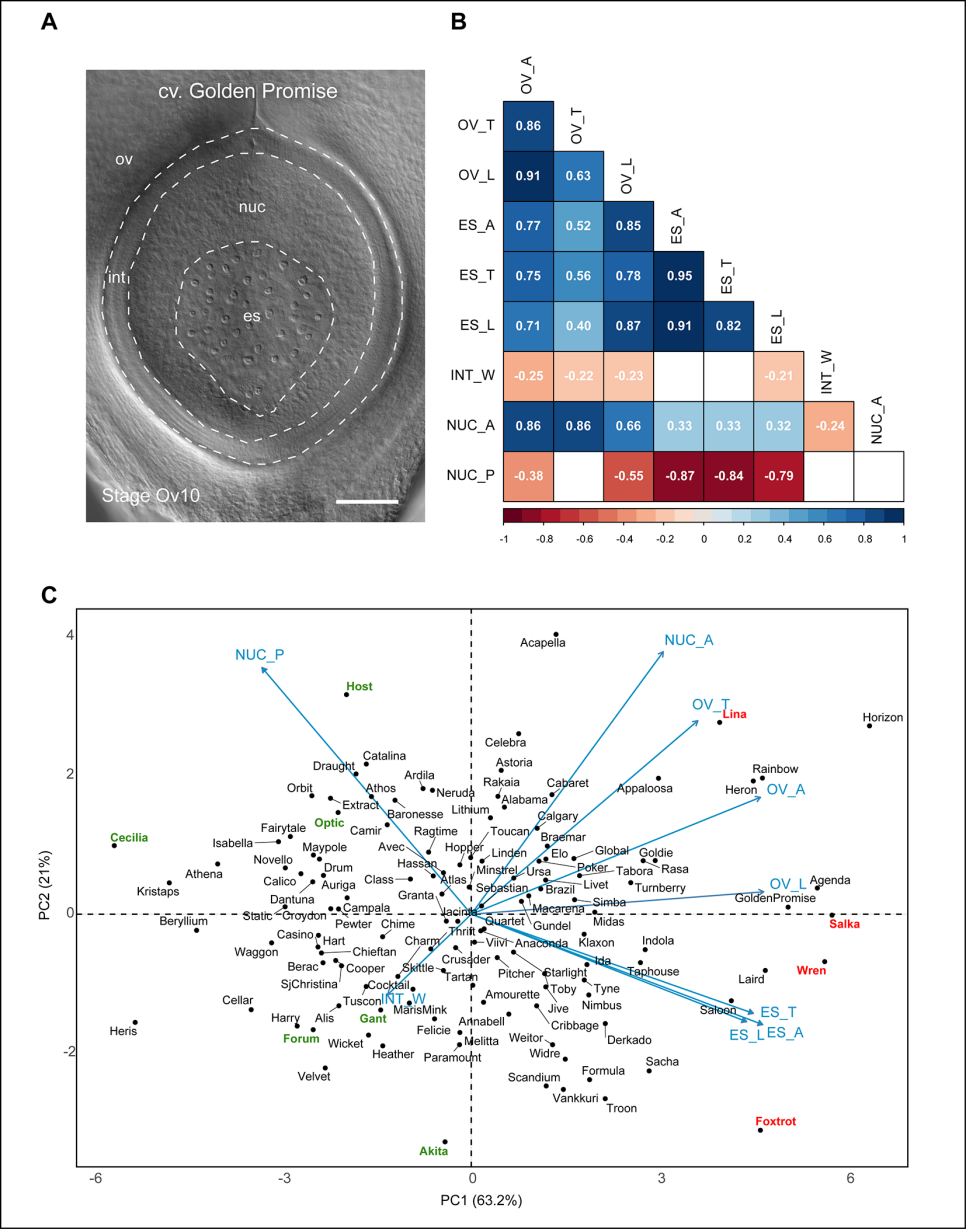
Relationships between different barley ovule traits at anthesis. **(A)** Anthesis ovule from Golden Promise showing the different regions used for measurements. Scale bar = 100 µm. **(B)** Heat map representing correlations between nine mature ovule traits measured in 127 genotypes of European two-row spring barley. Positive correlations are shaded blue, negative correlations are shaded red. Numbers within boxes represent the correlation coefficient (*r*) value. Both box color and *r* value are only shown for those with a p-value of < 0.05. OV_A, ovule area (µm^2^); OV_T, ovule transverse width (µm); OV_L, ovule longitudinal height (µm); ES_A, embryo sac area (µm^2^); ES_T, embryo sac transverse width (µm); ES_L, embryo sac longitudinal height (µm); INT_W, integument width (µm); NUC_A, nucellus area (µm^2^); NUC_P, nucellus proportion (%). **(C)** Principal component analysis of 127 genotypes of European two-row spring barley based on nine mature ovule traits. Key cultivars of interest to this study are highlighted in red (larger ovule features) or green (smaller ovule features).

Pearson correlation analysis was used to assess if overall variation in ovule morphology is a result of coordinated development of all ovule tissues, or whether growth of one tissue is more important (**Figures 4A**, **S4**). Nucellus area and ovule area showed a slightly higher correlation (*r* = 0.86, p < 0.001) than embryo sac area and ovule area (*r* = 0.77, p < 0.001; **Figure 4B**). In contrast, embryo sac area and nucellus area showed a significant but low correlation (*r* = 0.33, p < 0.001). Meanwhile, both embryo sac area and ovule area were negatively correlated with nucellus proportion (ovule area to nucellus proportion: *r* = -0.38, p < 0.001; embryo sac area to nucellus proportion: *r* = -0.87, p < 0.001). This suggests that while an increase in nucellus area reliably leads to larger ovule area, excessive embryo sac growth may achieve a similar outcome but at the expense of the nucellus. Bigger ovules were also more likely to have thinner integuments, as all ovule traits were negatively associated with integument width, particularly ovule area and nucellus area, although the correlation was low (ovule area to integument width: *r* = -0.25, p < 0.01; nucellus area to integument width: *r* = -0.24, p < 0.005).

The contribution of both embryo sac and nucellus traits to ovule size was reflected in the principal component analysis (PCA) plot (**Figure 4C**). The component indicators for embryo sac transverse width and longitudinal height (*r* = 0.82, p < 0.001) were closely positioned on the PCA plot in contrast to those for ovule transverse width and longitudinal height (*r* = 0.63, p < 0.001; **Figure 4B**). This suggests that variation in embryo sac area is more likely to be due to proportional variation in both transverse and longitudinal dimensions, whereas variation in ovule area may be due to independent changes in either direction. The PCA plot revealed an even spread of genotypes without obvious clustering, in addition to several clear outliers for each trait. Genotypes previously identified to have “extreme” phenotypes (**Table S2**) were located at the periphery of the PCA plot, which provides some insight into how variation in either nucellus area or embryo sac-related traits influence ovule area. For example, the large-ovule phenotypes of Golden Promise, Salka, and Wren appear to be driven by a combination of large nucellus and embryo sac traits. This differed from genotypes such as Host, which produced an “average” sized ovule with a relatively large nucellus area, and the above-average ovule area of Foxtrot, which was predominantly due to enlarged embryo sac traits. Other genotypes, such as Forum and Gant, produced an overall small-ovule phenotype due to smaller nucellus area. This indicates that although variability in embryo sac traits impacts ovule morphology, the overall “size” of the barley ovule is heavily dependent upon nucellus area.

To assess whether ovule features, in particular ovule area and nucellus area, were reproducible over subsequent generations, 10 genotypes were re-sown for analysis (**Table S3**, **Figure S5**). Genotypes were chosen that incorporated a range of ovule sizes including (from small to large); Cecilia, Forum, Gant, Akita, Optic, Host, Foxtrot, Wren, Salka, and Lina. Overall ovule area at maturity showed a Pearson correlation of 0.93 (p < 0.001) between subsequent generations, while nucellus area was slightly more variable but still significant at 0.79 (p < 0.01). Integument width also showed a significant correlation of 0.81 (p < 0.01). Embryo sac area showed a correlation of 0.56 but did not meet significance criteria. This confirms that in a subset of the population, ovule area, nucellus area and integument width show reproducible features across generations. This is an important finding for future studies since it suggests a genetic component influences variation in ovule development, and this might be investigated by quantitative genetic screens.

### Grain Traits Vary Between Genotypes and Share Relationships With Same-Generation Ovule Morphology

The variation in ovule measurements identified in the barley panel provided an opportunity to assess quantitative relationships between sub-ovule tissues and grain traits. Grain from plants grown in 2014 and 2015 were analyzed using a SeedCount^™^ SC4 instrument (Seed Count Australasia, Condell Park, Australia). Grain samples were sorted to focus only on genotypes showing evidence of good fill and at least 50 grain in at least 2 replicates. After filtering, 73 genotypes remained for comparison between years and with ovule phenotypes (**Table S4**).

Within each generation of grain, similar trends were observed. Grain width and thickness appeared to be the major indicators of grain weight compared to grain length (**Figure S6**). Similarly strong positive correlations were identified between grain weight and two-dimensional grain area (*r* = 0.71, p < 0.001 and 0.69, p < 0.001 within 2014 and 2015 grain, respectively). Despite being grown in glasshouses with similar environmental regimes, differences in sowing dates in 2014 and 2015 appeared to significantly impact fill; grain weight was 42.8 ± 0.99 in 2014 *vs*. 35.2 ± 0.56 in 2015 (t-test, p = 0.001). This is perhaps unsurprising given the later sowing date. However, several correlations were identified across the 2 years. For example, grain weight showed a small but significant correlation (*r* = 0.39, p < 0.001; **Figures S6**, **S7A**). A similar correlation was identified for grain width (*r* = 0.30, p < 0.01; **Figure S5**), in addition to slightly weaker correlations for grain thickness and area (**Figure S6**).

Grain traits were compared to ovule measurements from the same 73 genotypes (**Figures S6**, **S7**). No significant correlations were observed between grain morphology in 2014 and ovule morphology in progeny plants. In contrast, several significant correlations were identified between ovule morphology and features of the grain they produced (**Figures S6**, **S7**). Ovule transverse width, ovule area and nucellus area showed small but consistent negative correlations with all of the grain traits. For example, nucellus area showed a negative correlation with grain area (*r* = -0.37, p < 0.01; **Figure S7B**) and grain weight (*r* = -0.37, p < 0.01; **Figure S6**). The only positive correlation was observed between integument width and grain thickness (*r* = 0.35 p < 0.01; **Figure S7C**). Despite embryo sac area showing a strong correlation with ovule area in the same panel (*r* = 0.83 p < 0.001), no correlation was observed between any embryo sac measurement and grain measurement.

These effects appeared to be even more prominent when considering phenotypic extremes (**Figure S8**). Genotypes showing the largest nucellus area (n = 20) were compared to those showing the smallest nucellus area (n = 20; **Figure S8A**). As expected, these genotypes also showed a corresponding difference in overall ovule size (**Figure S8B**). In addition, grain weight (*t*-test, p = 0.01; **Figure S8C**) and grain area (*t*-test, p = 0.008; **Figure S8D**) were significantly reduced in those genotypes showing a larger nucellus and larger ovule. We considered that these differences in grain weight might be due to differences in fertility and the number of grain per spike. Mature spikes from at least 8 tillers of the 10 variable genotypes described above (Cecilia, Forum, Gant, Akita, Optic, Host, Foxtrot, Wren, Salka, and Lina) were scored for grain number and spike length. Analysis revealed differences in the number of grain per spike (from 18 to 24, average 21.6 ± 1.6) but these did not show any significant correlation with grain or ovule size. Hence, although the physiological basis for this variation in grain weight and size remains unknown, the results presented here are consistent with a pre-fertilization sporophytic ovule component that influences downstream features of grain development.

## Discussion

The plant ovule is a key reproductive organ that supports growth of the female gametophyte and establishes an environment for seed development. Previous studies in barley have focused on the role of the ovule nucellus around the time of fertilization and beyond (Radchuk et al., 2006; Thiel et al., 2008; Tran et al., 2014), revealing its role as a nutrient transfer tissue and identifying key genes that control its maturation and function (e.g. *Jekyll*; Radchuk et al., 2006). Despite this, little information is available regarding early stages of ovule and nucellus development in cereal monocots, or whether variation in nucellus growth affects downstream seed development. Tissue-specific components of fertility and seed development are relatively unexplored in cereal species, but may hold promise for future attempts to increase yield through modified breeding strategies, increased yield potential, or protection against stress (Whitford et al., 2013; Alqudah and Schnurbusch 2014; Brinton and Uauy 2018; Wilkinson et al., 2018).

This study examined the range of natural variation present in mature ovule phenotypes among a population of two-row spring barleys. Nine distinct stages of ovule development were identified by tissue clearing and morphological analysis, and these were aligned with previous staging studies in rice (Lopez-Dee et al., 1999; Itoh et al., 2005). Stages Ov2 to Ov4 describe the initiation of the germline lineage in the ovule, stages Ov5 to Ov8 incorporate mitotic divisions of the embryo sac, stages Ov9a and 9b show expansion/proliferation of ovule tissues, and stage Ov10 reflects reproductive maturity at anthesis. These ovule stages were aligned with the Waddington scale (Waddington et al., 1983), to simplify staging of ovule development in barley. A diagram showing the alignment of these scales is shown in **Figure S2**.

Data was collected from 150 genotypes at stage Ov10 to establish an “average” phenotype for the ovule at maturity and to identify genotypes showing variation in ovule morphology. It is generally reported that male and female reproduction in cereals is synchronized (Bennett et al., 1973; Kubo et al., 2013), thus the developmental stage of the anthers should reflect that of the less-accessible ovule. Here, developmental stage was predicted in two ways: (1) by assessing similarity of the anthesis pistil to that described in the Waddington scale, and (2) by determining whether the anthers of each floret were yellow in color and ready to release pollen. In some cases, pistil clearing revealed unexpectedly small ovule features, consistent with ovule immaturity. At the other extreme, some pistils contained an overly large ovule that appeared to have been fertilized (Diboll 1968; Engell 1994; Maeda and Miyake 1996, Maeda and Miyake, 1997; **Figure S3**). Collection of ovules at points before and after maturity, despite attempts to stage for reproductive maturity, may reflect sampling error. However, as anther and pistil phenotypes were used as a staging reference, we speculate that late male and female reproductive development are not perfectly synchronized in some two-row spring barley genotypes.

### Large Ovules in Barley Typically Contain an Enlarged Nucellus

As the megasporangium, or the tissue that ultimately gives rise to the female germline, the nucellus is a key component of ovule fertility. Nucellus area varied up to ± \29% in the barley panel, and was tightly coupled to overall ovule size. Despite this, as ovule size increased, the proportion of nucellus tended to decrease. The reason for this was embryo sac expansion, since embryo sac area showed a clear negative correlation with nucellus proportion. Hence, increased embryo sac and nucellus area may both drive increases in ovule area, but the expanding embryo sac increases in size at the expense of the nucellus. This might be facilitated by pre-fertilization degradation of nucellus cells adjoining the embryo sac, genotype-specific proliferation of antipodal cells and/or through mechanical compression of nucellus cells over time (Johri et al., 2013).

Despite the fact that nucellus development varies between species, the functional significance of intra- and inter-species variation in nucellus size has remained unclear (Rudall et al., 2005; Rudall et al., 2008; Endress 2011; Lu and Magnani 2018). In cereals, hypotheses suggest that a bigger nucellus might provide a larger repository of amino acids, carbohydrates, or hexose sugars that are required for the early stages of grain development (Wilkinson et al., 2018), but this has yet to be conclusively shown. The multilayered tissue may also act as a buffer that sustains female fertility during periods of abiotic stress (Saini and Aspinall, 1982; Saini et al., 1983). Alternatively, a larger nucellus may facilitate formation of an optimal environment for signaling during gametogenesis. Developmental signals such as phytohormones are transmitted through the nucellus (Cheng et al., 2006; Pagnussat et al., 2009) and contribute to gametogenesis prior to fertilization (Lora et al., 2017; Juranić et al., 2018). Moreover, studies of mutants that produce extra female germline-like cells in rice and *Arabidopsis* reveal a key difference in the competency of these ovule cells to enter meiosis. “Extra” germline cells in rice typically enter meiosis, but this is not the case in *Arabidopsis*. This may reflect a specific feature of the larger nucellus in rice and its ability to provide more stimulatory signals for germline development (Nonomura et al., 2003;Lora et al., 2019).

### A Link Between Nucellus Growth and Grain Development?

Correlation analysis in this study revealed small but significant negative relationships between ovule and grain phenotypes in the same generation, such that genotypes with a larger nucellus and ovule were more likely to produce smaller and lighter grain. In barley, the nucellus undergoes PCD and forms the nucellar projection, which functions as a transfer tissue facilitating movement of maternal nutrients to the developing embryo and endosperm (Thiel et al., 2008). Delayed PCD of nucellar cells dramatically reduces barley grain fill (Radchuk et al., 2006). Hence, one possible reason for the inverse relationship between nucellus size and seed weight is that a large nucellus may take longer to fully differentiate into a transfer tissue (i.e. the nucellar projection), thereby slowing down subsequent influx of nutrient into the fertilized embryo sac to support early endosperm divisions. Whether this relates exclusively to the syncytial phase or cellularization phase of grain development is unclear. In both rice and *Arabidopsis*, early syncytial stages of seed development play a critical role in seed size (Sundaresan 2005;Folsom et al., 2014). However, in barley at least, syncytial divisions appear to take place before maternal nutrient transfer pathways are fully established. Studies investigating the timing of nucellar PCD and differentiation suggest that flow of maternal nutrients into the fertilized embryo sac coincides with cellularization at around 5–6 days after pollination (Radchuk et al., 2006; Radchuk et al., 2010; Radchuk et al., 2012; also reviewed in Lu and Magnani 2018). Hence, the stage at which the embryo sac gains access to maternal nutrients is likely to be a factor influencing endosperm cellularization and subsequent seed size and weight (Weschke et al., 2003). A recent study in *Arabidopsis* provides some support for an antagonistic relationship between the nucellus and endosperm (Xu et al., 2016). Fertilization of the central cell in *Arabidopsis* cues degeneration of the nucellus *via* a series of MADS-box factors including *AGL62* and the B-sister gene *TT16*; these factors also act to repress nucellus growth and facilitate development of the chalazal endosperm. In the absence of *AGL62* function, the nucellus fails to degenerate and seed development aborts (Xu et al., 2016). Further examination of barley grain development will be required to distinguish when differences in weight and area appear, and to assess how this relates to differentiation of the nucellus.

The inverse relationship between nucellus size and seed weight appears somewhat distinct from other female traits examined in cereal species. For example, several studies identified a positive correlation between pistil size and yield traits, including grain size, in wheat and sorghum (Yang et al., 2009;Guo et al., 2015). This led to the hypothesis that floral nutrient allocation is a determinant of not only floral organ size but floral survival, and thus total yield (Guo et al., 2016). Although the relationship between pistil size and ovule size was not investigated here, grain number was examined in a subset of the population. No obvious correlation was identified to suggest that the variation in ovule development might be attributed to differences in floret fertility or grain number.

Other unanswered questions from this study relate to the reproducibility of the ovule-grain relationship, whether it can be uncoupled by different environmental conditions and whether the underlying genetic basis for this variation can be identified. Importantly the variation between genotypes was significant for all traits. In addition, analysis of ten genotypes in 2015 and 2016 indicated that sporophytic features of ovule development are relatively stable across consecutive generations, showing correlations of 0.79 to 0.93 (p < 0.01; **Figure S5**). This suggests there is likely to be a genetic component underlying ovule variation, which might be the focus of future genome wide association studies (GWAS). However, while the 2015 ovule features showed a relationship with features of 2015 grain, no significant relationship was identified with features of 2014 grain. This might be explained by different sowing dates, glasshouse conditions and/or day length. Genotype-specific differences in female fertility and ovule morphology have been identified after heat and water stress in wheat (Saini et al., 1983;Onyemaobi et al., 2018). In future studies it will be intriguing to investigate the response of this barley panel to periods of controlled stress, particularly in relation to the stability of ovule features and their impact on grain development.

### Integument Development Varies in Barley Ovules At Maturity

In general, growth of the integuments and seed coat has been proposed to set an upper limit to final seed size (Windsor et al., 2000; Nakaune et al., 2005; Adamski et al., 2009; Fang et al., 2012; Du et al., 2014; Li and Li 2015). In *Arabidopsis* for example, the cytochrome P450 genes KLUH/CYP78A5 (KLU) and CYP78A9 influence proliferation of integument cells and subsequently the cells of the seed coat, ultimately regulating ovule fertility and overall seed size (Ito and Meyerowitz 2000; Adamski et al., 2009;Sotelo-Silveira et al., 2013; Zhao et al., 2018). In wheat, silencing of *TaCYP78A5* (the wheat orthologue of *KLU*) was found to restrict seed coat cell proliferation and cause a 10% reduction in grain size (Ma et al., 2015). During barley ovule development, the two integuments grow to completely encapsulate the nucellus before stage Ov7/8. In this barley panel, integument width was found to be the least variable of the nine ovule traits measured. Genotypes with larger ovules tended to have thinner integuments, but no clear correlation was detected between integument width and seed area or weight. One limitation of the imaging method used in this study is the lack of information regarding the number of cells in each tissue type. These measurements would be required to determine whether features of the integuments, other than width alone, contribute to quantitative variation in ovule and seed development.

### Variable Ovule Phenotypes Provide Tools for Future Studies

Results from this study indicate that the size of mature ovules in barley is likely to be determined by a combination of factors such as nucellus proliferation, embryo sac expansion, and mechanical restriction *via* the integuments. How these features interact at the molecular and cellular level remains unclear, as does the link between sub-ovule features and grain size and weight. In future studies it may be possible to analyze these relationships at even greater resolution. For example, Mendocilla-Sato et al. (2017) recently reported a method for morphometric analysis of rice ovules that holds promise for analysis of cell shape, volume, and number. In addition, whole-mount clearing techniques such as ClearSee and PeaClarity (Kurihara et al., 2015; Palmer et al., 2015) are compatible with fluorescent markers and stains, providing avenues to track, visualize, and quantify individual cells and tissue types. Thus, in the future, similar data might be utilized to investigate the genetic architecture of barley ovule development, potentially highlighting candidate genes that contribute to female fertility, reproductive stress tolerance and grain morphology.

## Data Availability Statement

All datasets generated for this study are included in the article/**Supplementary Materials**.

## Author Contributions

MT, RB, and LW conceived the study. LW undertook the majority of experiments. TW contributed to data analysis and presentation. LW and XY contributed to development of staging schemes. LW and MT wrote the manuscript. All authors read and edited the manuscript.

## Funding

We acknowledge funding from the Australian Research Council (DP180104092) to MT.

## Conflict of Interest

The authors declare that the research was conducted in the absence of any commercial or financial relationships that could be construed as a potential conflict of interest. 

The handling editor declared a past co-authorship with one of the authors [MT].
