# Supplementary material for: Natural Variation in Ovule Morphology Is Influenced by Multiple Tissues and Impacts Downstream Grain Development in Barley (Hordeum vulgare L.)
Source: Front Plant Sci. 2019 Oct 31;10:1374. doi: 10.3389/fpls.2019.01374 (PMC6834768; doi:10.3389/fpls.2019.01374)
Supplement: Supplementary file 1 [file DataSheet_1.pdf]

Supplementary Tables

Table S1

Genotype	OV_A (μm²)	OV_T (μm)	OV_L (μm)	ES_A (μm²)	ES_T (μm)	ES_L (μm)	INT_W (μm)	NUC_A (μm²)	NUC_P (%)
Acapella	202777.7 ± 38935	499.1 ± 25.6	533.9 ± 19.7	46464.9 ± 9679.1	216.5 ± 4.8	279.5 ± 15.7	38.1 ± 2.5	156312.8 ± 30894.7	0.77 ± 0.024
Agenda	227499.2 ± 42467	483.4 ± 17.1	625.2 ± 24.7	75121.6 ± 13856.8	272.9 ± 11.6	367.9 ± 21.7	49.5 ± 2	152377.7 ± 32315.5	0.667 ± 0.043
Akita	159405.5 ± 21166.8	421.5 ± 5.4	500.8 ± 15.8	56263.6 ± 15762.1	242.9 ± 8.6	328.1 ± 16.2	52.2 ± 2.1	103142 ± 10601.4	0.653 ± 0.065
Alabama	188015.8 ± 22567.6	466.9 ± 10	543.3 ± 9.5	47066.8 ± 8037.7	233.3 ± 10.1	290.3 ± 8.7	48.1 ± 1.2	140949 ± 17452	0.75 ± 0.027
Alis	150012.3 ± 16751.1	432.3 ± 5.8	468.9 ± 15.9	44269.9 ± 9124.8	237 ± 13.1	266.1 ± 16.7	46.7 ± 0.8	105742.4 ± 9603.8	0.707 ± 0.037
Amourette	171066.4 ± 22210.5	435 ± 7.6	527.8 ± 12.7	53021.2 ± 11671.7	236.9 ± 9.4	320.1 ± 10.8	47.4 ± 0.6	118045.2 ± 12930.1	0.693 ± 0.038
Anaconda	175754.9 ± 24729.3	434.9 ± 12.6	544.4 ± 11.5	49135.6 ± 8349.3	225.1 ± 9.1	320.9 ± 11.7	46.6 ± 0.3	126619.3 ± 21639.9	0.719 ± 0.043
Annabell	169975.6 ± 17536.6	464.5 ± 23.1	514.9 ± 7	55868.4 ± 5977.1	246.6 ± 6.1	316 ± 6.7	49.6 ± 1.1	114107.2 ± 21672.8	0.666 ± 0.061
Appaloosa	211603.2 ± 24692	492 ± 11.6	576.9 ± 13.7	57511.7 ± 9111.2	260.2 ± 11	309.6 ± 15.7	49.5 ± 1.1	154091.6 ± 18946.2	0.728 ± 0.029
Ardila	180109.1 ± 18186.7	465.5 ± 8.2	519.5 ± 9.6	41395.2 ± 8153.9	214.4 ± 9.5	276.4 ± 8.2	49.7 ± 1.1	138714 ± 11530.8	0.772 ± 0.027
Astoria	190873.9 ± 16285	471.6 ± 5.3	543 ± 11.2	46474.6 ± 9324.7	224.7 ± 7.5	286.3 ± 12.1	47.5 ± 1.1	144399.3 ± 8488.4	0.759 ± 0.032
Athena	149659.1 ± 45201.7	412.8 ± 19	476.2 ± 26	32568.6 ± 18038.1	174.1 ± 15.8	254 ± 26.4	45.5 ± 1.3	117090.6 ± 31701.4	0.793 ± 0.07
Athos	160966.7 ± 27244.7	482.2 ± 49.1	497.9 ± 18.3	38129.9 ± 11155.5	201.4 ± 13.9	273 ± 17.2	44.7 ± 1.4	122836.7 ± 18501	0.766 ± 0.038
Atlas	171584.4 ± 32144.6	444.2 ± 18.4	530.7 ± 30.2	45944.1 ± 18271.2	207.3 ± 23.3	321.1 ± 27.1	46.4 ± 1.2	125640.3 ± 14108.7	0.742 ± 0.053
Auriga	161884.1 ± 38547.9	430.7 ± 15	503.2 ± 24.7	38298.6 ± 12018.4	191 ± 10	286.6 ± 18.1	48.8 ± 0.4	123585.5 ± 27274.8	0.768 ± 0.03
Avec	172691.2 ± 21624.7	447.1 ± 12.6	531.6 ± 10.2	44083.8 ± 8870.9	209.6 ± 4.2	312.6 ± 11.3	45.8 ± 1.8	128607.4 ± 22714.6	0.742 ± 0.055
Baronesse	171348.2 ± 28762.3	452 ± 13.7	514.5 ± 27.5	38308.1 ± 9522.4	197.4 ± 11.8	304.5 ± 16.8	45.3 ± 0.4	133040.1 ± 20688.9	0.778 ± 0.024
Berac	151120 ± 15047.7	419.8 ± 7.9	497.4 ± 23.9	38984.4 ± 8527.6	195.2 ± 3.7	310 ± 24.2	46.7 ± 0.5	112135.6 ± 9043.4	0.744 ± 0.037
Beryllium	138197.8 ± 15888.7	396.9 ± 11.7	484.3 ± 8.9	30462.7 ± 5597.7	174.6 ± 10.6	270.1 ± 10.4	44.9 ± 2.3	107735.1 ± 11329.2	0.781 ± 0.022
Braemar	187581.4 ± 11179.7	454.6 ± 10.1	562.9 ± 9.2	47076.2 ± 14935	239.7 ± 8.4	340 ± 19.3	47.5 ± 0.5	140505.2 ± 12090.8	0.751 ± 0.075
Brazil	182011.1 ± 23031.5	462.3 ± 11.3	542.3 ± 9.8	51241.6 ± 5545.8	242.9 ± 6.8	323.5 ± 8.4	45.5 ± 0.9	130769.6 ± 19270.8	0.717 ± 0.024
Cabaret	193191.6 ± 26708.1	466.6 ± 12.7	557.3 ± 16.3	48388.4 ± 10239.8	229.6 ± 7.9	321.9 ± 12.7	45.7 ± 0.8	144803.1 ± 19611.1	0.751 ± 0.033
Calgary	187296.8 ± 36417.7	472.4 ± 22	545.3 ± 22.5	49399.6 ± 10033.6	226.8 ± 13.2	325.5 ± 11	46.7 ± 1.2	137897.2 ± 27812.3	0.736 ± 0.021
Calico	156593.3 ± 21815.8	429.7 ± 10.5	495.7 ± 13.3	36313.5 ± 10750.5	186.6 ± 10	281.3 ± 10.2	46.6 ± 0.7	120279.8 ± 14262.8	0.772 ± 0.043
Camir	172567.8 ± 11254.8	461.2 ± 10	502.2 ± 6.1	39585 ± 4712.2	210.2 ± 6.7	286.9 ± 11.2	51.1 ± 0.5	132982.8 ± 12026.8	0.77 ± 0.032
Campala	162723.7 ± 30920	434.7 ± 11.6	503.4 ± 18.9	40636.5 ± 14579.3	201.7 ± 14.9	291.8 ± 14.6	49.5 ± 0.7	122087.3 ± 17067.7	0.759 ± 0.049
Casino	153786.7 ± 18049.9	435.5 ± 7.2	471.4 ± 8.3	40642.7 ± 6321.1	214.2 ± 6	278 ± 6.8	48.6 ± 0.7	113144 ± 14808.7	0.735 ± 0.031
Catalina	173326.2 ± 16742.5	461.7 ± 6.6	512.1 ± 13.5	35811.6 ± 10565.4	193.3 ± 10.8	286.7 ± 16.6	50.1 ± 0.8	137514.6 ± 12036.1	0.795 ± 0.049
Cecilia	136893.5 ± 27697.2	411.1 ± 17.7	440 ± 26.2	25194.4 ± 9330.6	158.1 ± 13	238.8 ± 24.3	46.1 ± 0.6	111699.2 ± 18406.4	0.821 ± 0.027
Celebra	193488.6 ± 15004.4	480 ± 7.8	533.3 ± 10	46708.3 ± 8401.9	229.2 ± 10.7	279.6 ± 13.1	43.5 ± 0.7	146780.3 ± 12442.5	0.759 ± 0.035
Cellar	148462 ± 34500.2	384 ± 26.2	485.2 ± 24.4	38385.3 ± 16361.6	200.2 ± 15.3	281.8 ± 21.2	49.8 ± 0.9	110076.7 ± 19948.7	0.752 ± 0.061
Charm	161985 ± 21394.4	421.9 ± 18.9	523.3 ± 8.4	45378.9 ± 10688.6	200.3 ± 18.5	327.1 ± 14.3	47.4 ± 2	116606.1 ± 11745.4	0.724 ± 0.038
Chieftan	155828.3 ± 12691.9	419.3 ± 4.5	511.5 ± 15.2	40089.8 ± 9515.4	196.7 ± 11.1	296.3 ± 19.9	50.8 ± 1.4	115738.5 ± 4680.8	0.746 ± 0.043

Chime	161668.1 ± 10602.1	434.8 ± 6.4	504.5 ± 10.5	43478.5 ± 6809.9	219.7 ± 7	288.9 ± 12.7	46.8 ± 0.7	118189.6 ± 7204.6	0.732 ± 0.031
Class	169898.4 ± 18278.3	436.2 ± 4.9	517.7 ± 20	43506.7 ± 13188.3	212.5 ± 18.5	300.6 ± 20.7	45 ± 0.3	126391.6 ± 6550.9	0.749 ± 0.053
Cocktail	163254.5 ± 25345	426.4 ± 10.7	525 ± 19.8	47006.1 ± 11979.9	215.3 ± 9.9	323.3 ± 18.3	49.6 ± 0.9	116248.3 ± 16431.4	0.715 ± 0.041
Cooper	155485.3 ± 25762.9	421.5 ± 8.5	504 ± 12.9	41315.4 ± 10263.4	205.8 ± 8.5	294 ± 11.6	48.6 ± 0.7	114169.8 ± 16507.1	0.737 ± 0.03
Cribbage	177799.4 ± 22963.1	443.1 ± 9.8	538.3 ± 12.5	57237.6 ± 10063.5	225.2 ± 4.3	358 ± 11.4	47.8 ± 0.7	120561.8 ± 15258.1	0.679 ± 0.029
Croydon	160034.6 ± 38047.1	437.1 ± 20.3	492.2 ± 36.1	41044.2 ± 15323.3	201.5 ± 12.1	277.7 ± 18.6	49.3 ± 0.5	118990.4 ± 23349.3	0.75 ± 0.033
Crusader	171243.3 ± 15637.8	444.1 ± 9.9	523.1 ± 9.8	48232.8 ± 12141	228.7 ± 13.4	317.5 ± 12.6	49.6 ± 0.6	123010.5 ± 9026	0.721 ± 0.054
Dantuna	158939.3 ± 22284.7	443.3 ± 11.3	482.3 ± 13.9	38076.7 ± 9948.8	198 ± 11.9	273.8 ± 14.1	49.6 ± 0.8	120862.5 ± 17061.1	0.762 ± 0.045
Derkado	186747.9 ± 22732.1	443 ± 8.2	565.1 ± 14.7	62685.7 ± 13968	252.9 ± 11.3	355.2 ± 16.3	49.3 ± 0.7	124062.2 ± 14565.2	0.667 ± 0.05
Draught	169698.7 ± 20772.6	455.8 ± 9.9	494.4 ± 9.3	36127.7 ± 3998	194.2 ± 4.6	282.4 ± 10.5	45.4 ± 0.7	133571.1 ± 17902.4	0.786 ± 0.017
Drum	160297.4 ± 21842.7	439.8 ± 9.2	491.5 ± 13.4	37227.3 ± 7495	195.9 ± 8.4	276.7 ± 10.9	47.5 ± 0.9	123070.1 ± 14843.9	0.77 ± 0.021
Elo	185090.6 ± 11378.9	458.4 ± 6.3	547.5 ± 7.2	50198.2 ± 5220.8	236.4 ± 3.5	329 ± 6.1	43.6 ± 0.5	134892.4 ± 9025.8	0.729 ± 0.022
Extract	164652.1 ± 14406.3	448.3 ± 6.9	497.6 ± 6.5	34479.2 ± 7094.9	188.9 ± 7.8	284 ± 8.3	46.7 ± 0.8	130172.9 ± 11537.1	0.791 ± 0.035
Fairytale	158521.2 ± 36092.7	440.4 ± 18.2	478.1 ± 21.3	35954.4 ± 13135.1	189.5 ± 17.3	264 ± 15.9	46.2 ± 1.6	122566.7 ± 24630.2	0.78 ± 0.046
Felicie	161461.4 ± 25734.5	430.3 ± 12.3	513.8 ± 10.3	49945.3 ± 10762.9	234.3 ± 8.8	305.8 ± 8.6	46.3 ± 0.7	111516.1 ± 20226.3	0.69 ± 0.048
Formula	179950.3 ± 16592.1	431.9 ± 7.1	570 ± 9.1	62091 ± 9557.1	247.6 ± 9.3	372.9 ± 10	50.7 ± 0.7	117859.4 ± 8938.8	0.657 ± 0.029
Forum	152996.3 ± 21356.1	398.6 ± 14.6	520.9 ± 15.2	43990.9 ± 15934.7	190.7 ± 22	295.7 ± 27.4	51.2 ± 1.1	109005.4 ± 16656.1	0.716 ± 0.084
Foxtrot	198862.9 ± 17717.3	449 ± 8.2	597.8 ± 10.5	77888.1 ± 8793.7	280.5 ± 7.8	406.6 ± 10.1	50.3 ± 1.3	120974.8 ± 13459.6	0.608 ± 0.032
Gant	158950.9 ± 39588.9	421.9 ± 12.8	511 ± 25.1	46619.9 ± 20650.8	209.9 ± 17	321.1 ± 19.9	50.4 ± 1.1	112331 ± 20229.6	0.719 ± 0.056
Global	192288.1 ± 17768.1	472.7 ± 12.1	551 ± 9.5	54115.5 ± 10936.5	224.5 ± 14.7	343.2 ± 11.4	47.8 ± 0.9	138172.5 ± 8588.4	0.721 ± 0.036
Golden Promise	216556.9 ± 14298.1	496 ± 10	590.7 ± 4.5	72314.9 ± 2290.7	281.7 ± 4	371.1 ± 3.8	48.7 ± 0.5	144242 ± 12135.2	0.665 ± 0.013
Goldie	204627.8 ± 14264.1	471.1 ± 12.4	572.7 ± 5.4	59582.5 ± 3252.3	245.4 ± 6.2	355.2 ± 9	47.4 ± 1.1	145045.3 ± 15604.8	0.707 ± 0.03
Granta	171594.5 ± 27088.2	428.8 ± 10.7	536.5 ± 14.9	47375.8 ± 11696.6	209.1 ± 9.6	322.9 ± 14	44.6 ± 1.5	124218.8 ± 18092.4	0.726 ± 0.034
Gundel	183395.7 ± 19038.4	444.8 ± 7.4	555.6 ± 10.2	50780.3 ± 10286.8	223.4 ± 9.9	341.2 ± 12.1	47.9 ± 0.8	132615.3 ± 9565.7	0.726 ± 0.033
Harry	143960.4 ± 15404.5	422.6 ± 7.6	463.9 ± 6.9	41114.3 ± 6085.6	206.4 ± 6	292.5 ± 5	47.6 ± 0.5	102846.1 ± 13370.9	0.713 ± 0.037
Hart	154955.7 ± 15849.2	435.8 ± 8.5	476.8 ± 10.4	40823.2 ± 5849.9	197.6 ± 6.1	288.3 ± 6.1	48 ± 0.7	114132.5 ± 14603.9	0.735 ± 0.036
Hassan	172938.6 ± 28604.2	440.3 ± 9.3	530.4 ± 14.2	44054.1 ± 10196.2	212.3 ± 7.2	311.7 ± 12.3	46.6 ± 0.5	128884.5 ± 21981.6	0.746 ± 0.04
Heather	155101.1 ± 9871.1	426 ± 4.9	498.2 ± 7.2	47387 ± 5030.5	217.8 ± 4.8	311.4 ± 6.4	49.5 ± 1.1	107714.1 ± 6795.3	0.695 ± 0.021
Heris	128009.3 ± 10397.5	386.8 ± 9.7	446.3 ± 5.7	32134.1 ± 2326.1	176.7 ± 4.5	251.7 ± 8.5	47.6 ± 0.5	95875.3 ± 10194.1	0.748 ± 0.024
Heron	219524.7 ± 33382.7	501.3 ± 19.3	584.6 ± 23.4	64910.8 ± 20208	264.5 ± 24.8	349 ± 24.3	43.7 ± 0.4	154613.9 ± 21015.5	0.71 ± 0.065
Hopper	176250.4 ± 2733.6	450.8 ± 1.5	522.3 ± 7.2	46541.8 ± 1841.5	222.4 ± 1.8	303.3 ± 12.1	44.9 ± 0.5	129708.7 ± 3117.5	0.736 ± 0.011
Horizon	241310.7 ± 48703.9	519.2 ± 24	607.2 ± 29.5	72158.9 ± 14691.2	268.8 ± 14.5	371.9 ± 24	43 ± 0.8	169151.8 ± 35130.8	0.7 ± 0.022
Host	174855.1 ± 22010.3	452 ± 12.2	513 ± 10.9	31372.8 ± 4873.2	170.3 ± 5.6	289.4 ± 8.5	44 ± 0.6	143482.3 ± 17884.2	0.821 ± 0.014
Ida	181751.8 ± 35204.9	452.9 ± 11.8	547.2 ± 18.9	57074.1 ± 16787.8	246.9 ± 11.3	359.6 ± 16.8	43.9 ± 0.8	124677.7 ± 20293.2	0.692 ± 0.043
Indola	192415.5 ± 25679.7	444.5 ± 8.8	607.5 ± 27.4	60038.8 ± 14732.8	233.4 ± 10.8	378.3 ± 21.3	45.2 ± 0.9	132376.7 ± 12874.2	0.692 ± 0.041
Isabella	155656.8 ± 32716.9	448.3 ± 16.4	458.7 ± 18.6	35115.4 ± 8884.8	196.4 ± 11.3	251.2 ± 12.1	46.1 ± 3.1	120541.4 ± 25453.2	0.774 ± 0.032

Jacinta	172285.4 ± 20494.2	444.2 ± 8.3	518 ± 9	47919 ± 11649.2	224.7 ± 9.5	312.6 ± 13.7	45.8 ± 0.6	124366.4 ± 14235.3	0.724 ± 0.05
Jive	177271.4 ± 48134.1	443.8 ± 19.5	528.6 ± 34.9	58553.1 ± 20528.7	241 ± 21.6	336.1 ± 22	42.1 ± 0.5	118718.3 ± 28599.7	0.676 ± 0.038
Klaxon	186547.5 ± 27052.2	451.5 ± 10.1	559.1 ± 16.8	57390.1 ± 14682.1	252 ± 13.2	331.4 ± 18.2	44.9 ± 0.6	129157.5 ± 16741.6	0.697 ± 0.059
Kristaps	141033.2 ± 16766.9	419.2 ± 7.9	451.1 ± 11.5	29149.3 ± 5087.3	172.1 ± 5.9	248.1 ± 10.6	48 ± 1.2	111883.9 ± 13941.6	0.793 ± 0.027
Laird	205907.9 ± 12201.4	478.3 ± 5.3	587.7 ± 9.0	72562.4 ± 9706.3	276.3 ± 6.1	379.3 ± 11.2	44.7 ± 0.6	133345.5 ± 8171.2	0.648 ± 0.036
Lina	218821.9 ± 23008.7	480.1 ± 14.6	618 ± 16.9	56061.7 ± 8325.2	245.8 ± 8.4	346.7 ± 12.2	41.8 ± 0.8	162760.2 ± 19881	0.743 ± 0.032
Linden	177054.9 ± 21240.3	440.6 ± 7.4	545.0 ± 8.8	45614.5 ± 7224.3	216.5 ± 5.5	322 ± 8.3	42 ± 0.4	131440.4 ± 20392.8	0.74 ± 0.047
Lithium	181754.7 ± 17181.1	453.5 ± 5.8	541.5 ± 6	45455.5 ± 6369	213.9 ± 3.9	317.5 ± 7.5	42.6 ± 0.5	136299.2 ± 14870.9	0.749 ± 0.031
Livet	185340 ± 16365.3	454.2 ± 12	555.3 ± 9.7	51007.7 ± 1965.7	228.7 ± 1.2	334.4 ± 7.1	45.2 ± 1.5	134332.3 ± 17738.9	0.722 ± 0.03
Macarena	179263.9 ± 28318	444.3 ± 18.2	539.9 ± 15.4	52068.4 ± 9691.3	228.3 ± 13.6	334.5 ± 6.4	39.1 ± 1.3	126477.7 ± 18769.5	0.715 ± 0.026
Maris Mink	159991.8 ± 16929.9	419.9 ± 12.5	503.8 ± 10.2	46703.7 ± 6238.6	220.4 ± 10.7	327.4 ± 9.3	45 ± 1.2	113288.1 ± 12547.9	0.708 ± 0.026
Maypole	159156.1 ± 31478.1	434.8 ± 16.4	486.1 ± 21	37549.7 ± 10722.4	192.2 ± 16	275.5 ± 11.4	44.5 ± 0.6	121606.4 ± 21810.1	0.767 ± 0.029
Melitta	162393.6 ± 22453.3	439.7 ± 8.6	494.5 ± 9.0	53423.8 ± 12408.2	236.3 ± 7.6	315.8 ± 8.4	43.8 ± 0.7	108969.8 ± 14518.4	0.673 ± 0.046
Midas	189991 ± 38369.1	458.8 ± 18.4	556.5 ± 29.1	56436.4 ± 12595.8	236 ± 12.6	353.3 ± 18.2	45.5 ± 0.9	133554.6 ± 25940.1	0.705 ± 0.011
Minstrel	175581.7 ± 17944.1	437.3 ± 6.4	541.7 ± 12.4	46950.9 ± 11014.2	215.7 ± 8.1	323.6 ± 15.3	44.4 ± 0.6	128927.6 ± 11016.1	0.742 ± 0.038
Neruda	177169.8 ± 19122.3	453.9 ± 8.1	531.6 ± 9.8	40801.9 ± 8715.7	200.3 ± 5.8	302.1 ± 10	44.3 ± 0.6	136367.9 ± 16535.1	0.769 ± 0.04
Nimbus	183543.5 ± 22967.5	435.6 ± 9.1	567.3 ± 10.9	58867.6 ± 13808.4	243.1 ± 11.8	357.5 ± 10.2	44.9 ± 1.1	124675.9 ± 14303.1	0.682 ± 0.046
Novello	154239.1 ± 16522.7	413.1 ± 5.5	505.6 ± 12.3	33330.3 ± 6097.1	171.7 ± 4.8	298 ± 10.8	43.9 ± 0.9	120908.9 ± 12418.2	0.785 ± 0.025
Optic	167477.1 ± 7342.4	439.9 ± 3.1	507.3 ± 7.4	35562.9 ± 3665.9	186.2 ± 6.3	294.3 ± 7.1	48.4 ± 0.6	131914.2 ± 5274.6	0.788 ± 0.016
Orbit	161883 ± 20556	451.7 ± 13.2	487.3 ± 8.5	33816.5 ± 4352.4	182.2 ± 7	281 ± 4.3	46.4 ± 1.2	128066.6 ± 18494.2	0.789 ± 0.026
Paramount	161360.3 ± 14144.7	413.7 ± 6.3	525.8 ± 15.4	51749.1 ± 13781.8	221.5 ± 14.4	342.1 ± 15.5	41.8 ± 0.9	109611.3 ± 2643.4	0.684 ± 0.061
Pewter	156268.5 ± 25146.1	423.6 ± 11	496.6 ± 10.3	37983.4 ± 5533.4	200.4 ± 6.5	298.6 ± 6.8	43.5 ± 0.5	118285.1 ± 21300.3	0.755 ± 0.026
Pitcher	172678.7 ± 11314.8	441.5 ± 5.6	532.4 ± 8.8	51923 ± 5262.5	234.8 ± 5.5	319.4 ± 7.8	44 ± 0.7	120755.7 ± 9224.1	0.699 ± 0.025
Poker	187373.3 ± 13702.9	455.1 ± 8.7	552.4 ± 11.7	50386.2 ± 9877.4	224.8 ± 9.1	334.2 ± 15.4	45.9 ± 0.9	136987.1 ± 11481.8	0.732 ± 0.045
Quartet	171754.4 ± 10072.4	450.7 ± 7.2	517.3 ± 7.1	48002.8 ± 10243.8	221.2 ± 14.3	348.8 ± 50.2	45.4 ± 1.1	123751.5 ± 7520.7	0.722 ± 0.048
Ragtime	170178.2 ± 26642.2	443.7 ± 14.7	519.3 ± 14.9	43434.9 ± 8846.7	202.3 ± 10.2	314.5 ± 13.8	41.2 ± 0.8	126743.3 ± 18976.8	0.746 ± 0.022
Rainbow	221620.1 ± 14500.2	488.6 ± 8.3	598.6 ± 8.1	65471.9 ± 8707.8	242.4 ± 6.9	375.2 ± 9.4	41 ± 1.2	156148.2 ± 8030.2	0.706 ± 0.025
Rakaia	183623.3 ± 16492.1	463.3 ± 8.6	521.5 ± 8.1	45941.3 ± 6966	213.7 ± 6.9	321.3 ± 6.8	40.9 ± 0.7	137682 ± 15001	0.749 ± 0.034
Rasa	194815.1 ± 36055.6	462 ± 18.7	582.6 ± 23.2	57361.3 ± 18451.3	230.1 ± 15.2	368.1 ± 26.5	39.6 ± 1.3	137453.8 ± 25536.5	0.707 ± 0.063
Sacha	183828.1 ± 29642.3	442.7 ± 9.5	580 ± 18.7	66886.2 ± 14705	255.3 ± 11.1	373.3 ± 13.4	45.5 ± 0.8	116941.9 ± 16409	0.639 ± 0.03
Salka	216504.3 ± 11109.6	476.6 ± 6.7	639.8 ± 8.9	72452.8 ± 7096.1	278.7 ± 7.5	396.9 ± 9.9	43.6 ± 0.4	144051.6 ± 11480.4	0.665 ± 0.032
Saloon	199385.6 ± 11367.7	456.7 ± 5.3	598.7 ± 7.1	70066.1 ± 9071.9	266.1 ± 6	382.3 ± 6.9	43.6 ± 0.5	129319.5 ± 10869.6	0.649 ± 0.039
Scandium	169544.2 ± 29001.2	437.8 ± 8.1	527.6 ± 17.7	60537.6 ± 17785.7	251.8 ± 11.9	350.2 ± 16.5	45.5 ± 0.5	109006.6 ± 14955.3	0.649 ± 0.055
Sebastian	177366.7 ± 12892	448.5 ± 10.9	535.3 ± 6.9	49655.6 ± 8576.6	214.8 ± 10	321.8 ± 19.5	46.7 ± 0.6	127711.1 ± 15658.4	0.719 ± 0.05
Simba	187989.2 ± 28982	470.9 ± 11.6	534.1 ± 15.6	55242.7 ± 13826	250.8 ± 15.7	332.8 ± 10.7	47.3 ± 0.8	132746.5 ± 17973.4	0.709 ± 0.041

Sj Christina	152980.5 ± 25152.2	420.8 ± 10.9	496.8 ± 12.3	42003.5 ± 10831.6	206 ± 9.4	295.5 ± 10.6	44.5 ± 0.9	110977.1 ± 16918.7	0.728 ± 0.041
Skittle	165843.4 ± 12277.1	429.9 ± 4.2	520.3 ± 8.2	47450 ± 8531.6	228.5 ± 8.8	316.8 ± 10.1	45.1 ± 0.6	118393.4 ± 9194	0.715 ± 0.04
Starlight	175739.2 ± 22371.3	440.8 ± 10.1	543 ± 14.5	52376.3 ± 15117.8	223.2 ± 14.7	348.9 ± 16	45.2 ± 0.5	123362.9 ± 10552	0.708 ± 0.056
Static	151390.8 ± 29178.8	412.4 ± 9.1	495.4 ± 18.9	34923.6 ± 6341.6	181.6 ± 6.1	301.5 ± 13.3	46.3 ± 0.8	118880.8 ± 24141.4	0.767 ± 0.024
Tabora	192978.2 ± 17445.3	450.4 ± 9.8	576.7 ± 8	53302 ± 5987.9	236.1 ± 6.7	334 ± 6.1	46.9 ± 1	139676.2 ± 12659	0.724 ± 0.016
Taphouse	192035.9 ± 29961.8	466 ± 9.4	556.7 ± 13.2	62763.8 ± 14264.2	253.1 ± 8.6	355.3 ± 10.3	46.1 ± 0.7	129272.1 ± 19587.2	0.675 ± 0.041
Tartan	166958.4 ± 19986.6	436 ± 7.8	524 ± 16.9	50389.5 ± 10818.3	231.5 ± 11.1	328.4 ± 11.1	44.7 ± 0.7	116568.9 ± 9242.8	0.702 ± 0.03
Thrift	163944.6 ± 13336.1	437.2 ± 6	516.9 ± 9.4	46666.3 ± 5608.5	224.4 ± 2.5	310.1 ± 9.1	44.5 ± 0.7	117278.2 ± 9335.9	0.716 ± 0.021
Toby	177399.8 ± 25090.6	449.9 ± 10.6	538.5 ± 11.6	55550.6 ± 12772.1	243 ± 11.1	338.7 ± 10.7	45.5 ± 0.7	121849.2 ± 15150.4	0.69 ± 0.041
Toucan	177561.6 ± 21683.1	444.7 ± 7.5	554.2 ± 13.5	45330.7 ± 9374.7	210.7 ± 6.9	320.3 ± 14	46.1 ± 0.6	132230.9 ± 14255.4	0.747 ± 0.033
Troon	178106.5 ± 15480.1	424 ± 7.7	580.2 ± 13.8	63375.3 ± 9998	253.2 ± 7.6	364 ± 12.6	46.5 ± 0.8	114731.2 ± 12625.3	0.644 ± 0.044
Turnberry	197208.8 ± 38990	458.2 ± 14.6	574.4 ± 34.1	58628.5 ± 24867.2	240.7 ± 25	355.1 ± 34.5	44.1 ± 0.9	138580.3 ± 19326.8	0.714 ± 0.082
Tuscon	154039 ± 30202.8	422.1 ± 10.7	502 ± 16.4	43908.6 ± 11936.7	218.2 ± 9.2	297.7 ± 13.6	44.9 ± 0.4	110130.4 ± 19497.2	0.718 ± 0.032
Tyne	183220.9 ± 29886.1	446.1 ± 14.2	547.9 ± 17.2	59202.6 ± 12854	257.8 ± 13.1	334.2 ± 11.3	44 ± 0.7	124018.3 ± 19111.5	0.679 ± 0.036
Ursa	180656.3 ± 16302.3	444.3 ± 7.1	551.3 ± 14	49858.8 ± 12481.4	227.8 ± 13.4	319.9 ± 16	42.7 ± 0.7	130797.5 ± 8571.4	0.727 ± 0.05
Vankkuri	172398.7 ± 53894.2	423.8 ± 32.9	549.8 ± 54.7	63755.6 ± 26969.7	240 ± 33.4	360.2 ± 48.7	42.5 ± 2.3	108643.1 ± 28393.1	0.652 ± 0.077
Velvet	143289.2 ± 27835.5	393.7 ± 9.1	491.1 ± 14.6	43372.5 ± 12077.3	210.7 ± 7.5	309.8 ± 13.7	42.4 ± 0.7	99916.7 ± 16250.7	0.702 ± 0.029
Viivi	170335.9 ± 32616.3	452.9 ± 19.9	506.4 ± 18.8	50929.4 ± 16704.8	234 ± 21.9	316.2 ± 17.2	44.6 ± 1	119406.6 ± 16508.7	0.707 ± 0.037
Waggon	146258.6 ± 24883.4	423.9 ± 9.1	471.6 ± 13.7	36657.3 ± 7066.1	203.9 ± 7.5	268.1 ± 8.6	46.3 ± 0.4	109601.3 ± 19976.5	0.748 ± 0.032
Weitor	169918.2 ± 21455.1	443.8 ± 8.8	521.7 ± 11	58549.7 ± 7184.3	256.3 ± 7.1	335.9 ± 8.5	41 ± 0.5	111368.5 ± 19181.1	0.653 ± 0.044
Wicket	152379.6 ± 17648.6	418.3 ± 7.3	499.9 ± 15.2	46210 ± 8823.6	222.2 ± 7.2	298.8 ± 12.4	46.6 ± 0.7	106169.6 ± 12074	0.698 ± 0.038
Widre	173886.7 ± 27079.8	448.3 ± 13.6	520.7 ± 12.7	60625.6 ± 11485.8	256 ± 12.4	349.8 ± 8.4	45.4 ± 0.9	113261.1 ± 18638.9	0.653 ± 0.041
Wren	210936.6 18576.4	488.7 ± 17.9	606.9 ± 11.7	76887.5 ± 15544.2	282 ± 11	387.8 ± 21.9	42.3 ± 0.9	134049.1 ± 15034.8	0.637 ± 0.056

Table S1: Average values for nine mature ovule traits in 127 genotypes of two-row spring barley. OV_A, ovule area; OV_T, ovule transverse width; OV_L, ovule longitudinal height; ES_A, embryo sac area; ES_T, embryo sac transverse width; ES_L, embryo sac longitudinal height; INT_W, integument width; NUC_A, nucellus area; NUC_P, nucellus proportion. Values show averages ± standard deviation.

Table S2

A

	OV_A	OV_T	OV_L	ES_A	ES_T	ES_L	INT_W	NUC_A	NUC_P
1	Heris	Cellar	Cecilia	Cecilia	Cecilia	Cecilia	Acapella	Heris	Foxtrot
2	Cecilia	Heris	Heris	Kristaps	Host	Kristaps	Macarena	Velvet	Wren
3	Beryllium	Velvet	Kristaps	Beryllium	Novello	Isabella	Rasa	Harry	Sacha
4	Kristaps	Beryllium	Isabella	Host	Kristaps	Heris	Rakaia	Akita	Troon
5	Velvet	Forum	Harry	Heris	Athena	Athena	Weitor	Alis	Laird
6	Harry	Cecilia	Alis	Athena	Beryllium	Fairytale	Rainbow	Wicket	Saloon
7	Waggon	Static	Casino	Novello	Heris	Alis	Ragtime	Heather	Scandium
8	Cellar	Athena	Waggon	Orbit	Static	Waggon	Paramount	Beryllium	Vankkuri
9	Athena	Novello	Athena	Extract	Orbit	Beryllium	Lina	Vankkuri	Weitor
10	Alis	Paramount	Hart	Static	Optic	Athos	Linden	Melitta	Widre
11	Berac	Wicket	Fairytale	Isabella	Calico	Dantuna	Jive	Forum	Akita
12	Static	Kristaps	Dantuna	Optic	Extract	Maypole	Wren	Scandium	Formula
13	Wicket	Chieftan	Beryllium	Catalina	Fairytale	Ardila	Velvet	Waggon	Salka
14	Sj Christina	Berac	Cellar	Fairytale	Forum	Drum	Vankkuri	Paramount	Golden Promise
15	Forum	Maris Mink	Maypole	Draught	Auriga	Croydon	Lithium	Cellar	Annabell
16	Casino	Sj Christina	Orbit	Calico	Maypole	Casino	Ursa	Tuscon	Derkado
17	Tuscon	Cooper		Waggon	Catalina	Acapella	Horizon	Sj Christina	Agenda
18	Novello	Akita		Drum	Draught	Celebra		Weitor	Melitta
19				Maypole	Berac	Orbit		Felicie	Taphouse
20				Pewter	Drum	Calico		Cecilia	Jive
21						Cellar		Kristaps	Cribbage
22						Draught		Berac	Tyne

B

	OV_A	OV_T	OV_L	ES_A	ES_T	ES_L	INT_W	NUC_A	NUC_P
1	Horizon	Horizon	Salka	Foxtrot	Wren	Foxtrot	Akita	Horizon	Cecilia
2	Agenda	Heron	Agenda	Wren	Golden Promise	Salka	Forum	Lina	Host
3	Rainbow	Acapella	Lina	Agenda	Foxtrot	Wren	Camir	Acapella	Catalina
4	Heron	Golden Promise	Indola	Laird	Salka	Saloon	Chieftan	Rainbow	Athena
5	Lina	Appaloosa	Horizon	Salka	Laird	Laird	Formula	Heron	Kristaps
6	Golden Promise	Wren	Wren	Golden Promise	Agenda	Indola	Gant	Appaloosa	Extract
7	Salka	Rainbow	Saloon	Horizon	Horizon	Rainbow	Foxtrot	Agenda	Orbit
8	Appaloosa	Agenda	Rainbow	Saloon	Saloon	Sacha	Catalina	Celebra	Optic
9	Wren	Athos	Foxtrot	Sacha	Heron	Formula	Cellar	Goldie	Draught
10	Laird	Lina	Golden Promise	Rainbow	Appaloosa	Horizon	Ardila	Cabaret	Novello
11	Goldie	Celebra	Laird	Heron	Tyne	Golden Promise	Cocktail	Astoria	Beryllium
12	Acapella	Laird	Heron	Vankkuri	Weitor	Rasa	Annabell	Golden Promise	Fairytale
13	Saloon	Salka	Rasa	Troon	Widre	Agenda	Crusader	Salka	Baronesse
14	Foxtrot	Global	Troon	Taphouse	Sacha	Troon	Dantuna	Host	Isabella
15	Turnberry	Calgary	Sacha	Derkado	Troon	Vankkuri	Heather	Alabama	Ardila
16	Rasa	Astoria	Appaloosa	Formula	Taphouse	Ida	Campala	Braemar	Calico
17		Goldie	Tabora	Widre	Derkado	Cribbage	Appaloosa	Tabora	Drum
18		Simba	Turnberry	Scandium	Klaxon	Nimbus	Agenda		Camir
19			Goldie	Indola	Scandium	Taphouse	Croydon		Acapella

20			Formula		Simba	Derkado	Derkado		Neruda
21						Goldie	Auriga		Auriga
22						Turnberry	Golden Promise		Maypole
23						Midas	Casino		Static
24									Athos

Table S2: Two-rowed spring barley genotypes exhibiting (A) small and (B) large variant phenotypes for nine mature ovule traits, as determined by exceeding standard deviation from average trait values. Genotypes are listed in descending order of variance from the average, i.e. smallest (A) and largest (B) genotypes are at the top of each table. OV_A, ovule area; OV_T, ovule transverse width; OV_L, ovule longitudinal height; ES_A, embryo sac area; ES_T, embryo sac transverse width; ES_L, embryo sac longitudinal height; INT_W, integument width; NUC_A, nucellus area; NUC_P, nucellus proportion.

Table S3

	2015								
Genotype	OV_A (µm2)	OV_T (µm)	OV_L (µm)	ES_A (µm2)	ES_T (µm)	ES_L (µm)	INT_W (µm)	NUC_A (µm2)	NUC_P (%)
Akita	159405.5 ± 21166.8	421.5 ± 5.4	500.8 ± 15.8	56263.6 ± 15762.1	242.9 ± 8.6	328.1 ± 16.2	52.2 ± 2.1	103142 ± 10601.4	65.3 ± 6.5
Cecilia	136893.5 ± 27697.2	411.1 ± 17.7	440.1 ± 26.2	25194.4 ± 9330.6	158.1 ± 13	238.8 ± 24.3	46.1 ± 0.6	111699.2 ± 18406.4	82.1 ± 2.7
Forum	152996.3 ± 21356.1	398.6 ± 14.6	520.9 ± 15.2	43990.9 ± 15934.7	190.7 ± 22.0	295.7 ± 27.4	51.2 ± 1.1	109005.4 ± 16656.1	71.6 ± 8.4
Foxtrot	198862.9 ± 17717.3	449.1 ± 8.2	597.8 ± 10.5	77888.1 ± 8793.7	280.5 ± 7.8	406.6 ± 10.1	50.3 ± 1.3	120974.8 ± 13459.6	60.8 ± 3.2
Gant	158950.9 ± 39588.9	421.9 ± 12.8	511.2 ± 25.1	46619.9 ± 20650.8	209.9 ± 17.0	321.1 ± 19.9	50.4 ± 1.1	112331 ± 20229.6	71.9 ± 5.6
Host	174855.1 ± 22010.3	452.1 ± 12.2	513.1 ± 10.9	31372.8 ± 4873.2	170.3 ± 5.6	289.4 ± 8.5	44.1 ± 0.6	143482.3 ± 17884.2	82.1 ± 1.4
Lina	218821.9 ± 23008.7	480.1 ± 14.6	618.2 ± 16.9	56061.7 ± 8325.2	245.8 ± 8.4	346.7 ± 12.2	41.8 ± 0.8	162760.2 ± 19881	74.3 ± 3.2
Optic	167477.1 ± 7342.4	439.9 ± 3.1	507.3 ± 7.4	35562.9 ± 3665.9	186.2 ± 6.3	294.3 ± 7.1	48.4 ± 0.6	131914.2 ± 5274.6	78.8 ± 1.6
Salka	216504.3 ± 11109.6	476.6 ± 6.7	639.8 ± 8.9	72452.8 ± 7096.1	278.7 ± 7.5	396.9 ± 9.9	43.6 ± 0.4	144051.6 ± 11480.4	66.5 ± 3.2
Wren	210936.6 ± 18576.4	488.7 ± 17.9	606.9 ± 11.7	76887.5 ± 15544.2	282 ± 11.0	387.8 ± 21.9	42.3 ± 0.9	134049.1 ± 15034.8	63.7 ± 5.6

	2016									
Genotype	OV_A (µm2)	OV_T (µm)	OV_L (µm)	ES_A (µm2)	ES_T (µm)	ES_L (µm)	INT_W (µm)	NUC_A (µm2)	NUC_P (%)	Grain/ Spike
Akita	165955.6 ± 14489.4	529.4 ± 27.6	422.4 ± 15.1	51402.2 ± 11033.8	339.5 ± 30.7	220.9 ± 25.5	47.4 ± 2.0	114553.3 ± 8732.6	69.2 ± 4.6	21.0 ± 1.7
Cecilia	173164.8 ± 17537.8	543.6 ± 32.6	439.9 ± 18.2	57167.4 ± 11662.7	360.3 ± 28.4	234.8 ± 29.6	45.6 ± 1.9	115997.3 ± 8054.6	67.2 ± 3.9	21.4 ± 1.3
Forum	166443.4 ± 24234.8	513.2 ± 45.3	438.7 ± 23.3	44776.6 ± 11770.1	314.4 ± 41.0	207.3 ± 22.9	49.4 ± 1.9	121666.7 ± 15151.6	73.3 ± 4.3	22.5 ± 1.6
Foxtrot	199438.3 ± 6716.4	569.0 ± 27.3	466.6 ± 12.1	63588.7 ± 12113.7	364.9 ± 38.3	255.9 ± 27.7	49.1 ± 1.9	135849.6 ± 9719.7	68.2 ± 5.4	23.1 ± 1.7
Gant	166737.7 ± 12759.8	528.2 ± 27.1	436.6 ± 12.9	49747.1 ± 8486.7	339.2 ± 27.9	220.2 ± 22.6	50.7 ± 2.1	116990.5 ± 6807.9	70.3 ± 3.4	24.4 ± 2.5
Host	187580.4 ± 21195.8	560.9 ± 36.5	456.6 ± 21.3	57771.0 ± 13899.8	378.9 ± 35.1	235.9 ± 34.0	45.2 ± 1.8	129809.3 ± 9706.4	69.5 ± 4.4	21.2 ± 2.8
Lina	207965.3 ± 10990.1	605.7 ± 22.2	469.8 ± 12.4	75469.2 ± 8034.8	414.5 ± 27.5	262.6 ± 18.6	43.4 ± 1.9	136294.7 ± 16006.8	65.6 ± 8.4	20.3 ± 3.3
Optic	170128.1 ± 14725.8	553.5 ± 31.9	420.6 ± 14.3	56387.1 ± 9032.8	357.8 ± 26.6	233.3 ± 25.3	46.4 ± 2.3	113741.0 ± 9779.4	66.9 ± 3.7	20.6 ± 3.3
Salka	204043.6 ± 14286.0	599.9 ± 31.6	476.6 ± 13.4	69212.6 ± 10901.6	390.5 ± 33.0	266.5 ± 23.7	45.9 ± 1.6	134831.0 ± 8060.7	66.2 ± 3.7	18.7 ± 1.8
Wren	201539.6 ± 14002.6	612.3 ± 24.7	454.9 ± 13.2	71935.8 ± 9460.01	406.3 ± 22.4	264.2 ± 27.0	46.3 ± 2.1	129603.8 ± 8478.9	64.3 ± 3.0	22.4 ± 1.8

Table S3: Average values for nine mature ovule traits among ten variable barley genotypes grown in 2015 and 2016. OV_A, ovule area (µm^2); OV_T, ovule transverse width (µm); OV_L, ovule longitudinal height (µm); ES_A, embryo sac area (µm^2); ES_T, embryo sac transverse width (µm); ES_L, embryo sac longitudinal height (µm); INT_W, integument width (µm); NUC_A, nucellus area (µm^2); NUC_P, nucellus proportion (%). The number of grain per spike was determined from 2016 plants. Values show averages ± standard deviation.

Table S4

	2015					2014				
**Cultivar**	**G_W (mg)**	**G_L (mm)**	**G_Wi (mm)**	**G_T (mm)**	**G_A (mm2)**	**G_W (mg)**	**G_L (mm)**	**G_Wi (mm)**	**G_T (mm)**	**G_A (mm2)**
Aapo	37.3	8.3	3.1	2.5	18.3	42.2	9.6	3.1	2.8	19.2
Acapella	31.8	7.2	2.9	2.3	16.1	52.2	9.9	3.4	2.2	22.6
Agenda	39.8	7.1	3.1	2.5	17.1	16.7	9.2	3.1	2.6	18.2
Akita	43.8	8.8	3.2	2.6	20.7	64.3	9.6	3.3	3.0	24.1
Alis	30.7	7.0	2.9	2.2	15.6	28.7	9.6	2.8	2.2	17.8
Alliot	31.1	9.3	3.0	2.3	19.3	47.6	9.4	3.2	2.4	19.5
Amourette	39.9	7.2	3.1	2.4	17.8	45.1	9.8	3.2	2.5	21.3
Ardila	34.5	6.8	3.0	2.4	16.0	39.0	9.1	3.1	2.3	19.4
Athos	32.4	7.9	3.0	2.4	17.3	36.2	9.8	3.2	2.7	21.4
Atlas	34.7	7.1	2.9	2.4	16.0	28.6	9.7	3.0	2.2	19.2
Brazil	35.1	8.5	3.0	2.4	18.8	27.3	9.5	2.8	2.3	18.6
Camir	31.6	6.9	3.0	2.4	15.9	35.0	8.3	3.0	2.5	16.3
Campala	36.1	7.1	3.0	2.5	15.8	35.2	8.9	2.9	2.2	18.0
Catalina	39.8	8.0	3.1	2.5	18.2	54.5	8.5	3.0	2.7	24.3
Cecilia	37.3	7.4	3.0	2.4	16.8	44.8	9.3	3.2	2.4	21.6
Cellar	41.4	7.3	3.2	2.5	17.8	44.0	10.1	3.2	2.3	21.7
Chariot	35.4	9.5	2.7	2.4	19.2	33.3	9.6	3.3	2.5	20.6
Charm	35.3	7.1	3.0	2.4	16.6	31.0	9.2	3.1	2.4	17.1
Class	38.3	9.6	3.1	2.5	21.3	47.9	9.9	3.4	2.7	22.6
Colada	41.8	8.6	3.2	2.6	19.6	53.4	9.9	3.3	2.5	21.5
Cribbage	39.9	9.7	3.2	2.5	20.7	37.8	9.6	3.3	2.8	20.5
Croydon	34.0	7.2	3.0	2.3	16.6	50.0	9.2	3.4	2.9	20.5
Crusader	35.7	7.7	2.9	2.2	17.4	36.2	10.2	3.1	1.8	20.0
Delibes	40.3	10.3	3.1	2.6	22.8	43.7	9.4	3.0	2.5	20.0
Draught	27.1	7.2	3.1	2.3	16.8	47.9	9.2	3.4	2.7	21.3
Drum	36.9	7.1	3.1	2.4	16.9	50.5	10.0	3.4	2.6	23.4
Elo	24.3	6.3	2.8	2.2	13.1	36.2	9.1	2.9	2.5	16.3
Fairytale	33.6	7.4	3.1	2.4	16.3	42.9	8.9	3.0	2.7	17.0
Felicie	36.6	7.9	3.0	2.3	17.8	43.1	9.5	3.2	2.5	20.7
Formula	38.0	7.2	3.1	2.4	17.6	45.4	10.1	3.3	2.3	23.8
Forum	29.5	8.2	2.8	2.4	17.4	30.8	9.8	2.6	2.0	18.3
Foxtrot	40.0	7.8	3.2	2.5	18.5	55.0	10.2	3.4	3.0	21.6
Gant	33.1	6.8	3.0	2.4	15.7	40.2	9.9	3.2	2.5	21.5
Global	35.3	6.8	3.2	2.4	16.3	43.9	8.9	3.4	2.6	19.7
Golden Promise	26.0	6.5	2.8	2.2	14.0	36.3	8.9	3.3	2.5	19.5
Hana	30.3	6.3	3.1	2.2	14.9	40.0	9.0	3.0	2.5	17.0
Harry	36.4	9.0	3.2	2.3	20.3	49.6	10.2	3.2	2.7	22.5
Hart	39.0	10.3	3.0	2.4	22.0	51.1	9.3	3.3	2.7	19.8
Heather	32.9	8.5	3.0	2.3	18.6	33.3	8.7	3.1	2.7	17.4

Helmi	32.7	6.5	3.0	2.3	15.0	36.0	9.9	3.0	2.3	19.4
Heron	33.7	6.7	3.1	2.5	15.8	47.1	9.3	3.4	2.5	21.6
Hindmarsh	25.1	6.3	2.8	2.1	13.5	50.0	9.9	3.3	2.9	20.2
Host	36.6	7.7	3.1	2.3	17.3	40.3	8.7	3.1	2.6	19.4
Ida	33.8	7.2	2.9	2.2	16.8	44.6	10.5	3.4	2.3	23.3
Indola	35.0	8.7	3.0	2.2	19.0	39.1	9.0	3.1	2.3	18.0
Isabella	37.1	7.6	3.0	2.5	16.4	55.0	9.7	3.8	2.8	23.4
Jacinta	35.4	8.7	2.9	2.3	17.9	45.3	9.3	3.1	2.5	19.6
Jive	41.2	8.8	3.2	2.4	19.9	44.3	9.5	3.1	2.7	21.1
Keops	36.4	6.4	3.2	2.5	15.3	37.6	9.5	3.1	2.3	20.5
Klaxon	38.8	8.4	2.8	2.3	17.9	44.7	9.6	3.1	2.7	20.0
Kristaps	35.9	8.3	3.0	2.3	18.1	35.0	9.5	3.1	2.5	18.7
Laird	30.8	7.0	3.0	2.3	16.3	39.5	8.7	3.2	2.5	19.5
Linden	46.5	8.9	3.1	2.4	20.1	62.3	10.9	3.3	3.0	23.5
Lithium	30.3	9.1	2.8	2.1	18.6	45.2	9.8	3.4	2.5	22.7
Livet	36.2	8.3	2.9	2.2	18.4	31.1	9.3	2.7	2.4	16.0
Maypole	37.8	7.2	3.0	2.4	16.9	52.1	10.4	3.4	2.6	23.6
Midas	41.0	8.6	3.0	2.5	19.7	50.6	9.1	3.4	2.6	21.0
Mikado	35.5	7.2	2.8	2.2	17.2	41.0	9.5	2.9	2.5	18.3
Quench	39.5	9.9	3.2	2.3	20.7	45.0	9.4	3.2	2.7	20.8
Saana	30.9	7.8	3.1	2.3	17.6	34.3	9.4	3.3	2.2	19.9
Scandium	30.3	8.3	2.8	2.3	16.8	46.8	9.9	3.3	2.7	21.3
Tabora	33.5	7.6	3.1	2.3	17.4	43.6	9.5	3.0	2.7	18.6
Tankard	33.2	7.3	3.0	2.4	16.5	47.3	8.9	3.4	2.7	19.9
Tartan	36.2	7.3	3.0	2.5	17.0	47.3	10.2	3.3	2.6	22.5
Thrift	36.3	8.0	3.1	2.4	18.6	50.0	9.5	3.3	2.9	20.0
Toucan	30.2	8.3	2.9	2.3	17.3	48.9	9.8	3.4	2.7	22.7
Troon	36.1	7.5	3.0	2.4	17.3	49.4	9.8	3.3	2.7	20.4
Tyne	26.2	7.3	2.7	2.1	15.3	38.1	9.5	3.0	2.3	19.2
Velvet	36.6	7.9	2.9	2.3	18.1	30.0	10.1	2.8	2.3	19.1
Waggon	42.5	9.7	3.5	2.6	23.6	44.6	9.6	3.2	2.8	18.8
Weitor	33.2	7.4	2.9	2.3	16.6	42.3	9.5	3.1	2.6	19.7
Wicket	47.0	7.8	3.2	2.7	19.0	59.8	9.8	3.7	3.1	23.3
Wren	23.1	7.5	2.7	2.0	14.9	37.1	9.7	2.8	2.6	17.3

Table S4: Grain traits measured in 73 genotypes of European two-row spring barley. G_W, average grain weight (mg); G_L, average grain length (mm); G_W, average grain width (mm); G_T, average grain thickness (mm); G_A, average grain area (mm^2).
