# Supplementary material for: Natural Variation in Ovule Morphology Is Influenced by Multiple Tissues and Impacts Downstream Grain Development in Barley (Hordeum vulgare L.)
Source: Front Plant Sci. 2019 Oct 31;10:1374. doi: 10.3389/fpls.2019.01374 (PMC6834768; doi:10.3389/fpls.2019.01374)
Supplement: Supplementary file 2 [file DataSheet_2.pdf]

Supplementary Figures

Figure S1

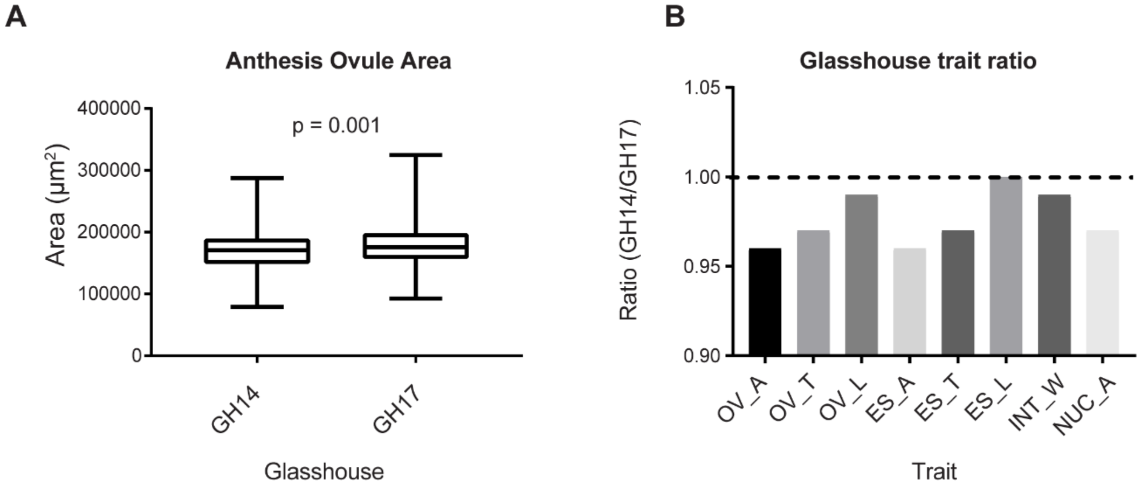

Figure S1: Variation in ovule development between glasshouses in 2015. (A) Mature (Stage Ov10) ovule area in Glasshouse 14 and 17. Bars show the trait distribution from maximum to minimum. (B) Ratio of ovule phenotypes in GH14/GH17. Labels as per Figure 1.

Figure S2

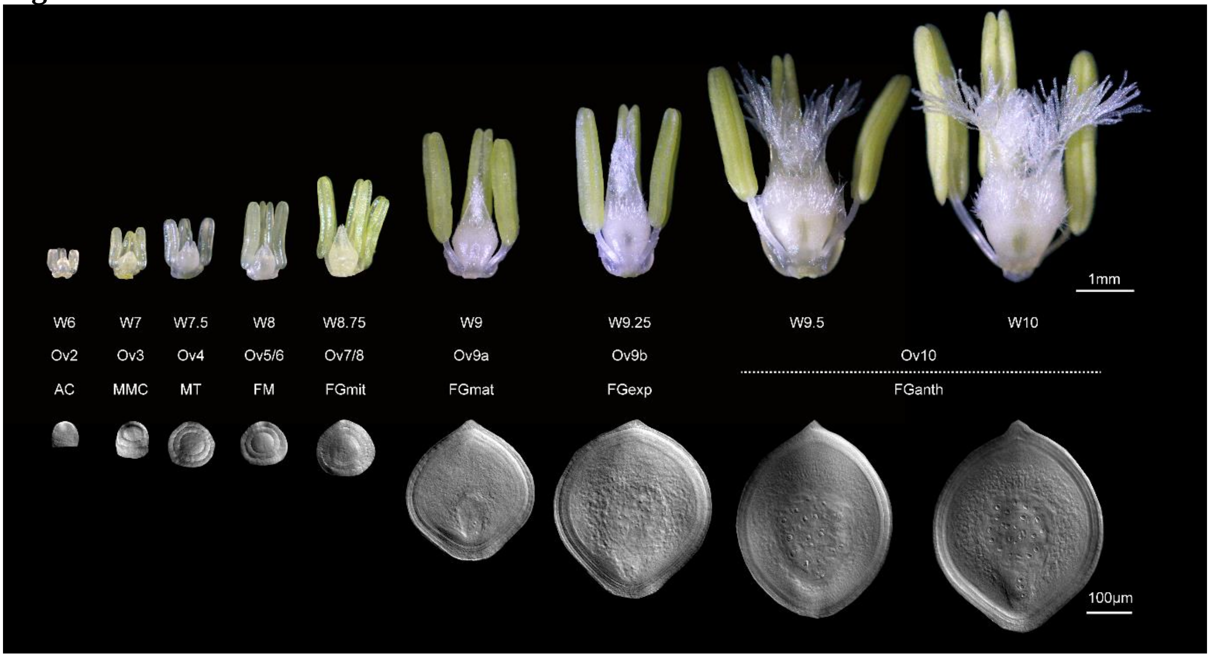

Figure S2: Alignment of Waddington stages of barley pistil development with stages of ovule development. Waddington stages are described in Waddington et al (1983). Ovule stages were determined by comparison to stages reported in rice (See Figure 1 legend; Itoh et al., 2005). Germline stages were assigned according to the identity of cells present within the ovule. AC, archesporial cell; MMC, megaspore mother cell; MT, meiotic tetrad; FM, functional megaspore; FGmit, female gametophyte mitosis; FGmat, mature female gametophyte; FGexp, expanding female gametophyte; FGanth, anthesis female gametophyte.

Figure S3

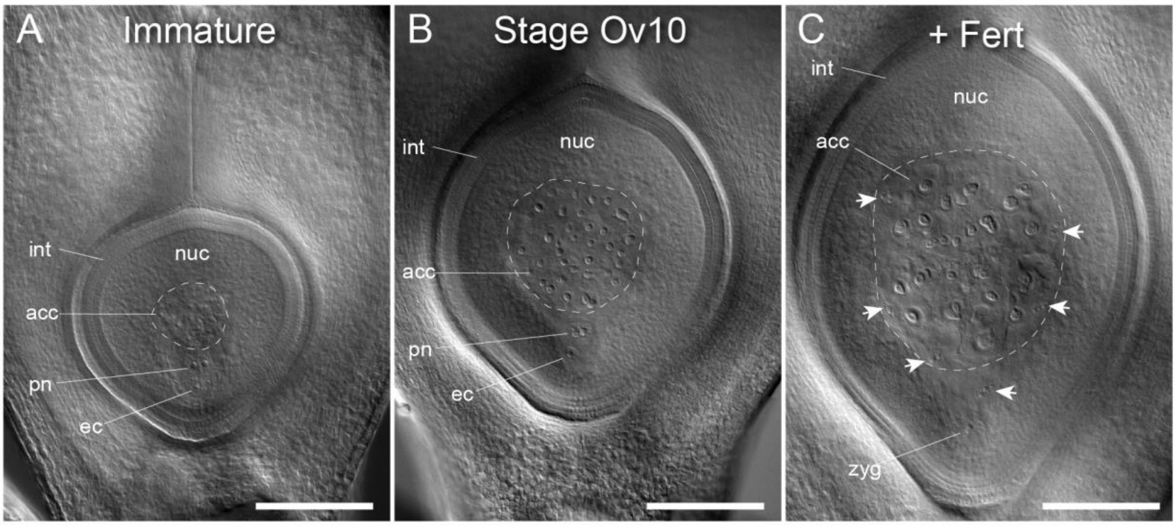

Figure S3: Variable phenotypes in barley ovules collected at anthesis. (A) Immature ovule (cv. Widre). (B) Mature, unfertilised ovule (cv. Scandium). (C) Fertilised ovule (cv. Scandium). acc, antipodal cell cluster; ec, egg cell nucleus; int, integument; pn, polar nuclei; nuc, nucellus; zyg, zygote. Arrowheads indicate small clusters of endosperm nuclei; dashed line indicates bounds of antipodal cell cluster. Scale bars = 200μm.

Figure S4

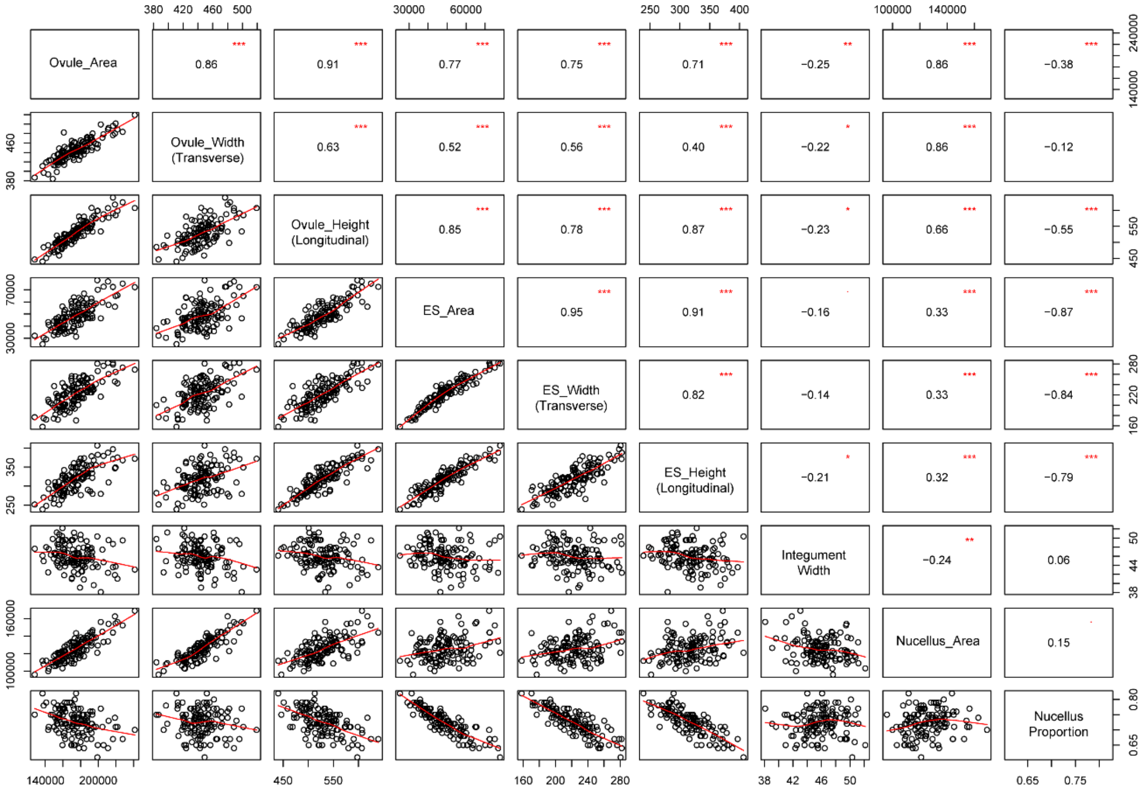

Figure S4: Correlation plots showing relationships between nine mature ovule traits in 127 barley genotypes at maturity (stage Ov10). Numbers within boxes represent Pearson's correlation coefficient (*r*) values. Significance is indicated as: * = p <0.05; ** = p < 0.01; *** = p < 0.001. Ovule_Area (µm^2); Ovule_Width (Transverse) (µm); Ovule_Height (Longitudinal) (µm); ES_Area (embryo sac area; µm^2); ES_Width (Transverse) (µm); ES_Height (Longitudinal) (µm); Integument Width (µm); Nucellus_Area (µm^2); Nucellus Proportion (NUC_P; %).

Figure S5

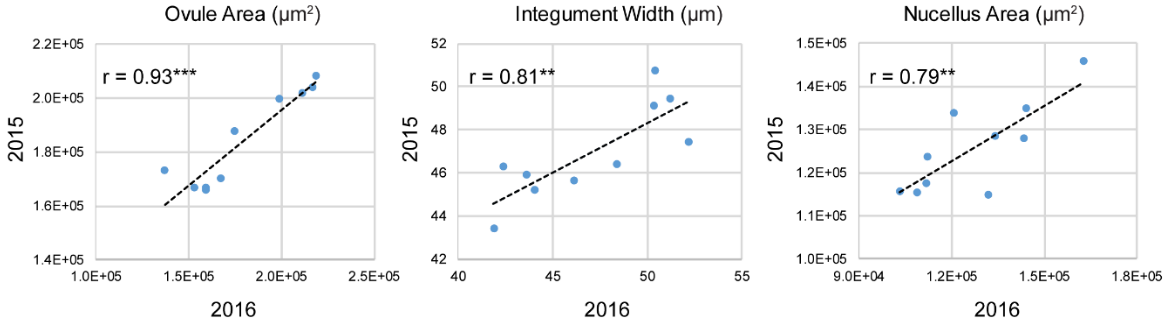

Figure S5: Reproducibility of selected mature ovule measurements for 10 genotypes in 2015 and 2016. From left to right panels show comparisons for ovule area, integument width and nucellus area. Correlation coefficient (*r*) values are shown, while significance is shown as ** = $p < 0.01$, *** = $p < 0.001$.

Figure S6

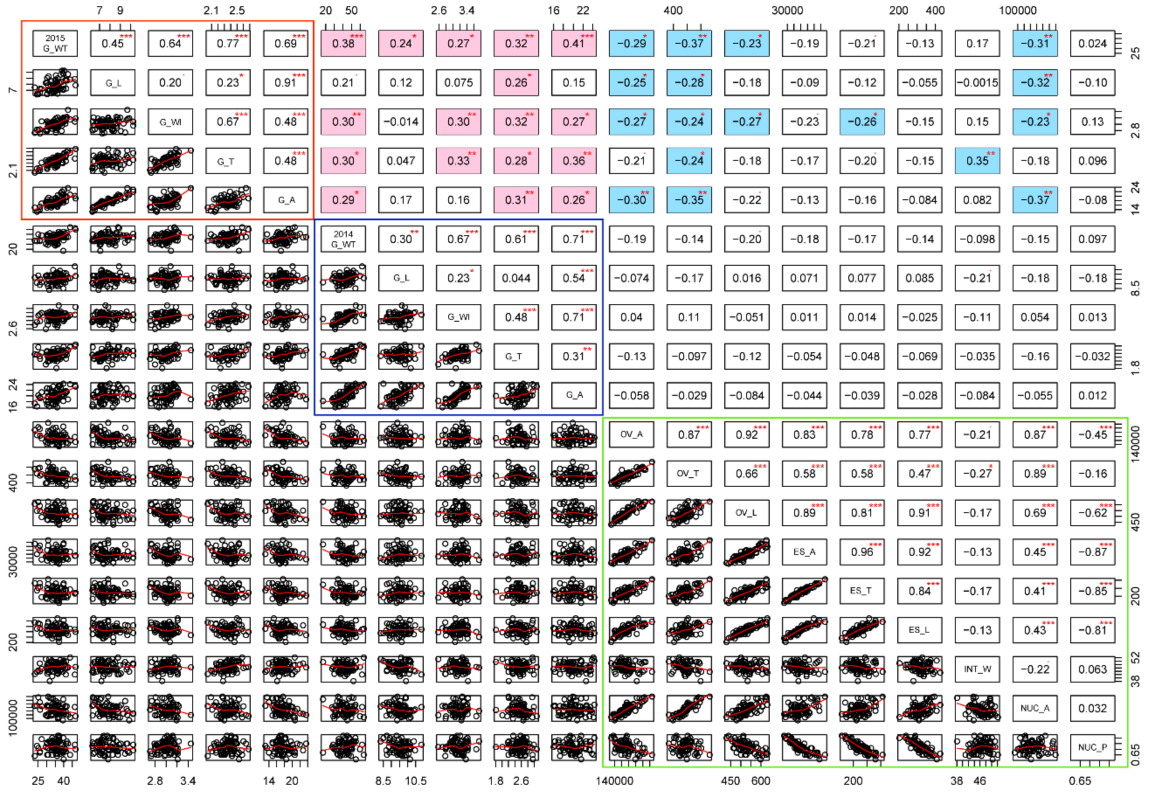

Figure S6: Correlation plots showing relationships between grain traits (2014 and 2015) and nine mature ovule traits in 73 genotypes of European two-row spring barley. Numbers within boxes represent correlation coefficient (*r*) values. Significance is shown as · = p <0.1, * = p <0.05; ** = p < 0.01; *** = p < 0.001. G_WT, grain weight (mg), G_L, grain length (mm); G_WI, grain width (mm); G_T, grain thickness (mm); G_A, average grain area (mm^2); OV_A, ovule area (μm^2); OV_T, ovule transverse width (µm); OV_L, ovule longitudinal height (µm); ES_A, embryo sac area (μm^2); ES_T, embryo sac transverse width (µm); ES_L, embryo sac longitudinal height (µm); INT_W, integument width (µm); NUC_A, nucellus area (μm^2); NUC_P, nucellus proportion (%). The red outline highlights 2015 grain measurements, the blue outline highlights 2014 grain measurements and the green outline highlights 2015 ovule measurements. Pink shading highlights significant correlations between grain measurements from 2014 and 2015, while blue shading highlights correlations between ovule and grain traits in 2015)

Figure S7

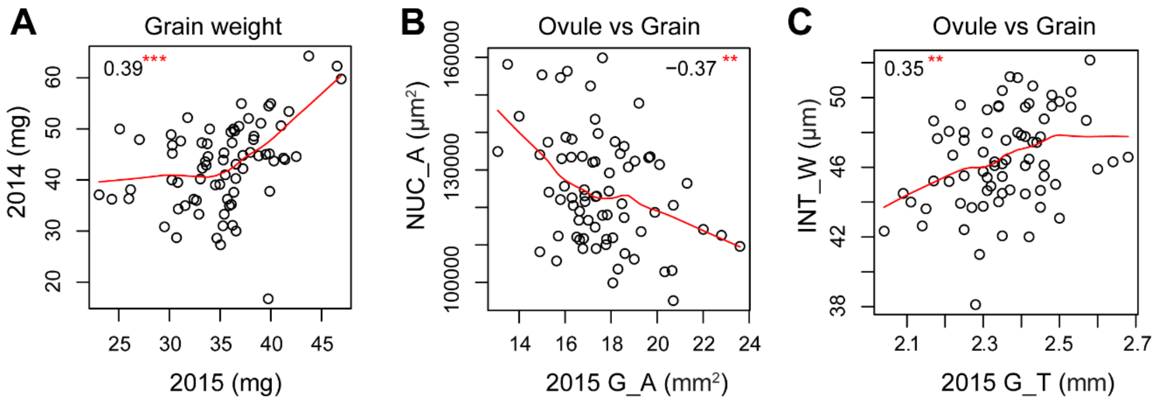

Figure S7: Correlations between grain and mature ovule measurements. (A) Grain weight comparison between 2014 vs 2015. (B) Nucellus area vs grain area (2015). (C) Integument width vs grain thickness (2015). Numbers within boxes represent correlation coefficient (*r*) values. Significance is shown as ** = $p < 0.01$; *** = $p < 0.001$.

Figure S8

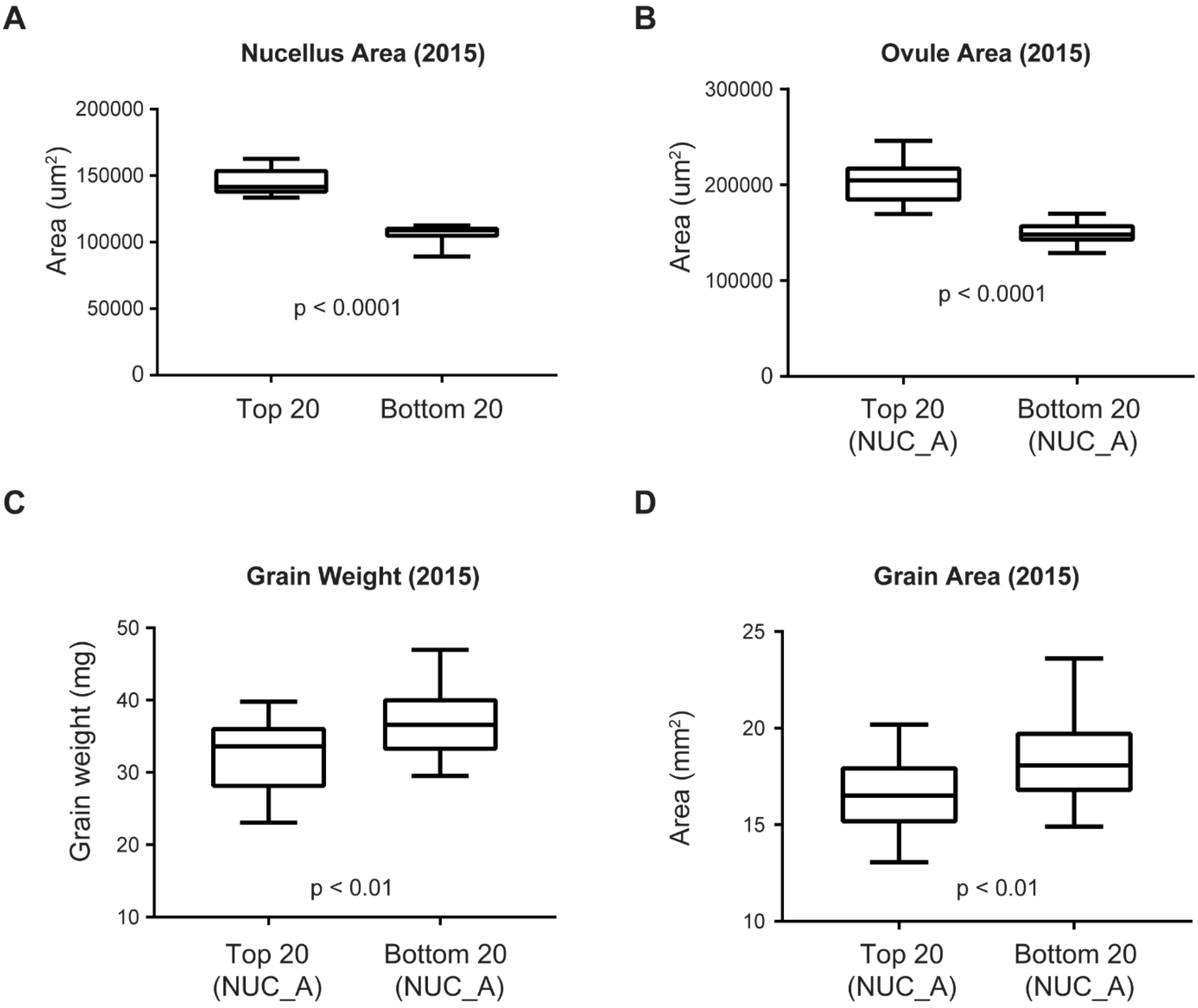

Figure S8: Comparisons between grain and mature ovule measurements in the top 20 and bottom 20 genotypes distinguished based upon nucellus area. (A) Variation in nucellus area between the 20 largest and 20 smallest extremes. (B) Variation in ovule area in the same genotypes as A. (C) Variation in grain weight in the same genotypes as A. (D) Variation in grain area in the same genotypes as A.
